# Multimodality Imaging-Based Characterization of Regional Material Properties in a Murine Model of Aortic Dissection

**DOI:** 10.1038/s41598-020-65624-7

**Published:** 2020-06-08

**Authors:** Matthew R. Bersi, Víctor A. Acosta Santamaría, Karl Marback, Paolo Di Achille, Evan H. Phillips, Craig J. Goergen, Jay D. Humphrey, Stéphane Avril

**Affiliations:** 1 Department of Biomedical Engineering, Yale University, New Haven, CT USA; 2 Department of Biomedical Engineering, Vanderbilt University, Nashville, TN USA; 3 Mines Saint-Etienne, University of Lyon, University Jean Monnet, INSERM, Saint-Etienne, France; 4 Weldon School of Biomedical Engineering, Purdue University, West Lafayette, IN USA; 5 Vascular Biology and Therapeutics Program, Yale School of Medicine, New Haven, CT USA

**Keywords:** Biophysics, Engineering

## Abstract

Chronic infusion of angiotensin-II in atheroprone (*ApoE*^−/−^) mice provides a reproducible model of dissection in the suprarenal abdominal aorta, often with a false lumen and intramural thrombus that thickens the wall. Such lesions exhibit complex morphologies, with different regions characterized by localized changes in wall composition, microstructure, and properties. We sought to quantify the multiaxial mechanical properties of murine dissecting aneurysm samples by combining *in vitro* extension-distension data with full-field multimodality measurements of wall strain and thickness to inform an inverse material characterization using the virtual fields method. A key advance is the use of a digital volume correlation approach that allows for characterization of properties not only along and around the lesion, but also across its wall. Specifically, deformations are measured at the adventitial surface by tracking motions of a speckle pattern using a custom panoramic digital image correlation technique while deformations throughout the wall and thrombus are inferred from optical coherence tomography. These measurements are registered and combined in 3D to reconstruct the reference geometry and compute the 3D finite strain fields in response to pressurization. Results reveal dramatic regional variations in material stiffness and strain energy, which reflect local changes in constituent area fractions obtained from histology but emphasize the complexity of lesion morphology and damage within the dissected wall. This is the first point-wise biomechanical characterization of such complex, heterogeneous arterial segments. Because matrix remodeling is critical to the formation and growth of these lesions, we submit that quantification of regional material properties will increase the understanding of pathological mechanical mechanisms underlying aortic dissection.

## Introduction

The development of vascular diseases, such as abdominal aortic aneurysm and thoracic aortic aneurysm and dissection, is often characterized by localized changes in wall composition and microstructural organization that lead to compromised tissue function. In particular, damage and degradation of elastin, loss of smooth muscle cells or their functionality, remodeling of collagen, local accumulations of glycosaminoglycans, and intramural thrombus can all alter material properties of the vascular wall. Given the temporal limitations of clinical data, animal models are a useful tool for unraveling potential roles of biochemomechanical factors in aneurysmal development^1^, the risk of dissection or rupture^2^, and the biomechanical role of intramural thrombus^1,3,4^. The ability to precisely excise and study vessels at various stages of disease severity allows for the generation of relationships between local wall composition and mechanical properties and can provide insight into conditions that may promote disease progression^5^.

Chronic infusion of angiotensin-II (AngII) in male hyperlipidemic apolipoprotein-E null (*ApoE*^−/−^) mice is a widely used mouse model that has a high incidence of lesion formation in the suprarenal abdominal aorta (SAA) and ascending thoracic aorta (ATA)^6,7^. The damaged SAA was first interpreted as an abdominal aortic aneurysm^8^, but is now recognized as an aortic dissection that precedes aneurysm formation and often contains intramural thrombus^9,10^. Indeed, recent data using synchrotron-based imaging confirms prior observations that SAA lesions often initiate as a medial tear near ostia of suprarenal branches^11,12^. Retrograde propagation of this initiating tear can create a false lumen with varying degrees of severity and intramural thrombus, though the biomechanical factors determining the extent of false lumen formation remain unknown^13–15^. Hence, these vascular lesions are structurally complex and often contain separate regions of blood flow and intramural thrombus within a false lumen bounded by a nonuniformly thickened aortic wall^16^.

Although diverse studies have advanced our molecular and biological understanding of this mouse model^4,17–19^, questions regarding the mechanical mechanisms of dissection formation and the impact of the development and maturation of intramural thrombus on the evolving biomechanics of the aortic wall remain outstanding^15^. The relative difficulty of studying such mechanical mechanisms in murine vessels has led to the need for specialized equipment and methods, especially given the marked geometric and material heterogeneities in and near the dissections. Of importance herein, the presence of a false lumen and intramural thrombus in dissected vessels prevents one from using traditional biaxial testing^20^. In traditional approaches, vessels are often assumed to behave as thin-walled structures with homogenous properties through the vessel wall – both of which are not applicable for dissected regions of the aorta with intramural thrombus. Indeed, it was for this reason that SAA dissections were avoided in our prior study using this mouse model^2^. Note, therefore, that we have previously developed a novel approach to characterize the local nonlinear, anisotropic mechanical properties of murine arteries by combining biaxial extension-distension testing, panoramic digital image correlation (pDIC), and an inverse method based on the principle of virtual power^5,21^. Moreover, we have recently extended this approach by including optical coherence tomography (OCT) to assess full-field wall thickness to complement the full-field surface strains obtained via pDIC. This multimodality based approach proved useful in characterizing spatial variations of radially averaged material and structural properties in ascending thoracic aortic aneurysms from multiple mouse models^22^.

In the current study, we extend our method further by processing OCT images using a digital volume correlation (DVC) technique, thus enabling direct measurements of strain fields across thick-walled, dissected vessels that arise in the SAA of AngII-infused *ApoE*^−/−^ mice. Herein, we describe the overall methodology and report representative results from three mice that represent a range of vascular geometries and thrombus morphologies across the spectrum of these complex lesions. Local values of material stiffness and elastically stored energy are derived across both the dissected wall and the intramural thrombus. Finally, identified material properties are compared with constituent area fractions obtained from histological analyses to infer structure-property relations.

## Results

### Dissection morphology and structure

The dissected samples used in this study (denoted M1, M2, M3, and M4) were also used previously to perform complementary hemodynamic simulations^16^. Briefly, AngII infusion significantly increased blood pressure and led to localized medial ruptures and dissection between the media and the adventitia in each of the SAAs that was partially filled with thrombus. Samples were previously ordered by increasing dissection size, with no thrombus in M1 and the thickest thrombus in M4^16^. As the primary goal of this study was to develop and evaluate a new multimodality technique to reconstruct transmural material properties in thick-walled samples including thrombus, we have focused only on the dissected samples with thrombus (i.e., M2, M3 and M4); results for sample M1 are included in Fig. S1.

**Figure 1:**
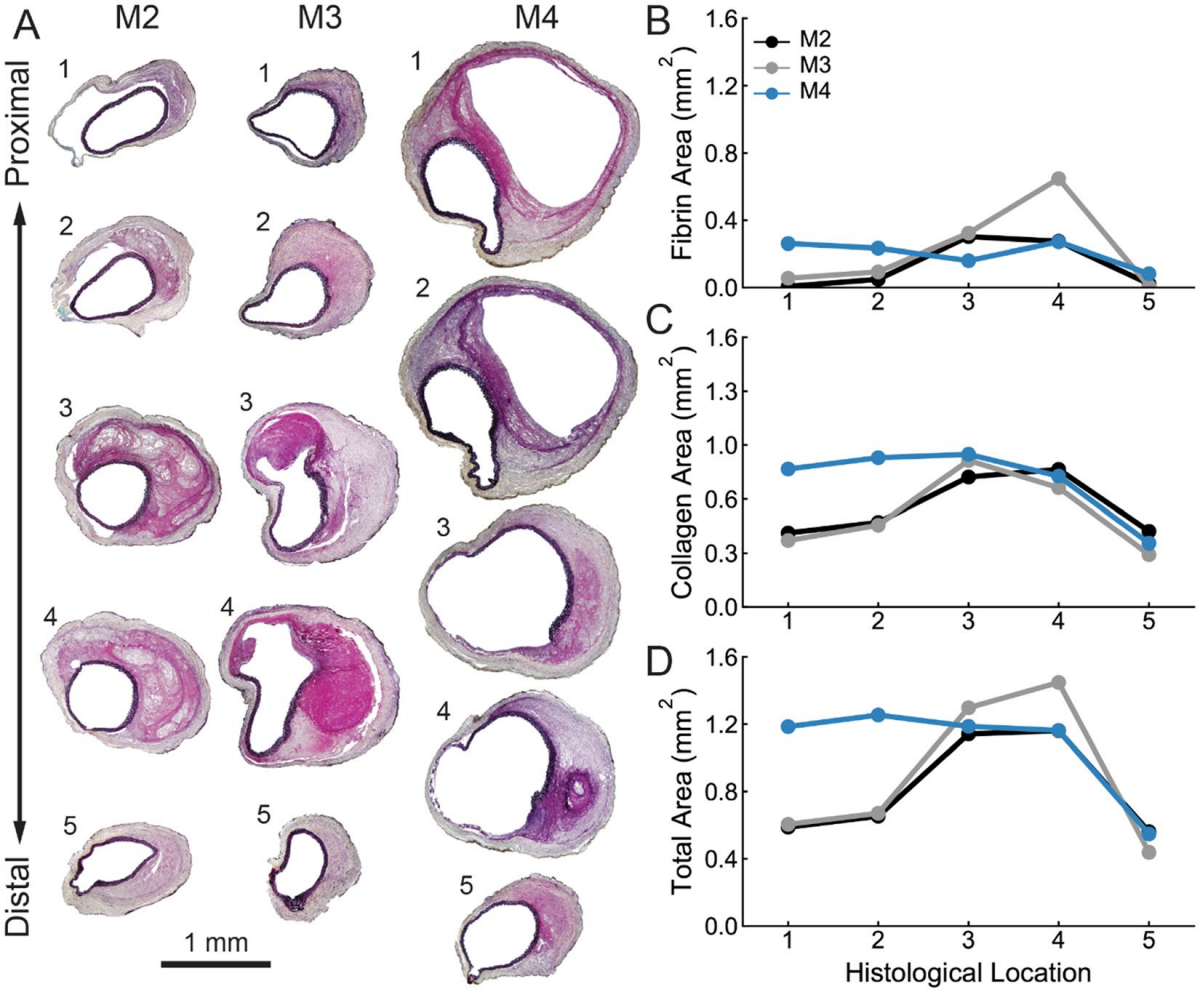
Histological analysis of aortic dissection samples. (**A**) Movat’s Pentachrome (MOV) stained images at 5 locations ordered from proximal to distal along the axis of each dissected sample (M2, M3, and M4) illustrates the geometric and microstructural variation between samples. Quantitative colorimetric analysis revealed spatial variations in (**B**) fibrin area, (**C**) collagen area, (**D**) and total wall area (excluding lumen and false lumen areas) both within and between samples.

The complex geometries of dissected samples were mirrored by complex intramural distributions of wall constituents. Histological sectioning along the length of samples M2, M3, and M4 highlights the significant variation in structure and composition (Fig. 1). Movat’s Pentachrome staining revealed localization of thrombus (or fibrin; red/pink), collagen (yellow/brown), and elastin (black) in each cross-section. Representative images of colorimetric segmentation and quantification of fibrin, collagen, and elastin are included in Fig. S2. The lesions from samples M2, M3, and M4 all presented with a false channel with freely flowing blood, which was confined to a small zone in M2 (Section 4; Fig. 1A) and M3 (Sections 3 and 4; Fig. 1A) but extended almost the whole length of the dissection in M4 (Sections 1–4; Fig. 1A). Quantification of the area of the wall occupied by fibrin (Fig. 1B) and collagen (Fig. 1C) revealed axial variations between samples. Indeed, M3 had the largest fibrin area of all samples (Section 4) and M4 had the largest collagen area of all samples at Sections 1 and 2. Interestingly, all samples had similar amounts of collagen in the distal half of the samples, consistent with retrograde thrombus accumulation or growth. Though M4 had a larger area at Sections 1 and 2, the total wall area was similar between samples at Sections 3–5 (Fig. 1D). A single centrally located cross-section was used for histological analysis of specimen M1 since there was no apparent pathology (see Fig. S1).

It is interesting that a significant fraction of the thrombotic regions – particularly in sample M2 – stained yellow-grey in Fig. 1A, indicating that fibrin had already begun to be replaced by collagen only ~7 days since the dissection initiated^4^. Note, too, that the medial layer is severed and retracted in the middle sections in specimens M3 and M4, consistent with an entrance site that allowed blood to enter the wall and create the false lumen, which is much more extensive in M4.

**Figure 2:**
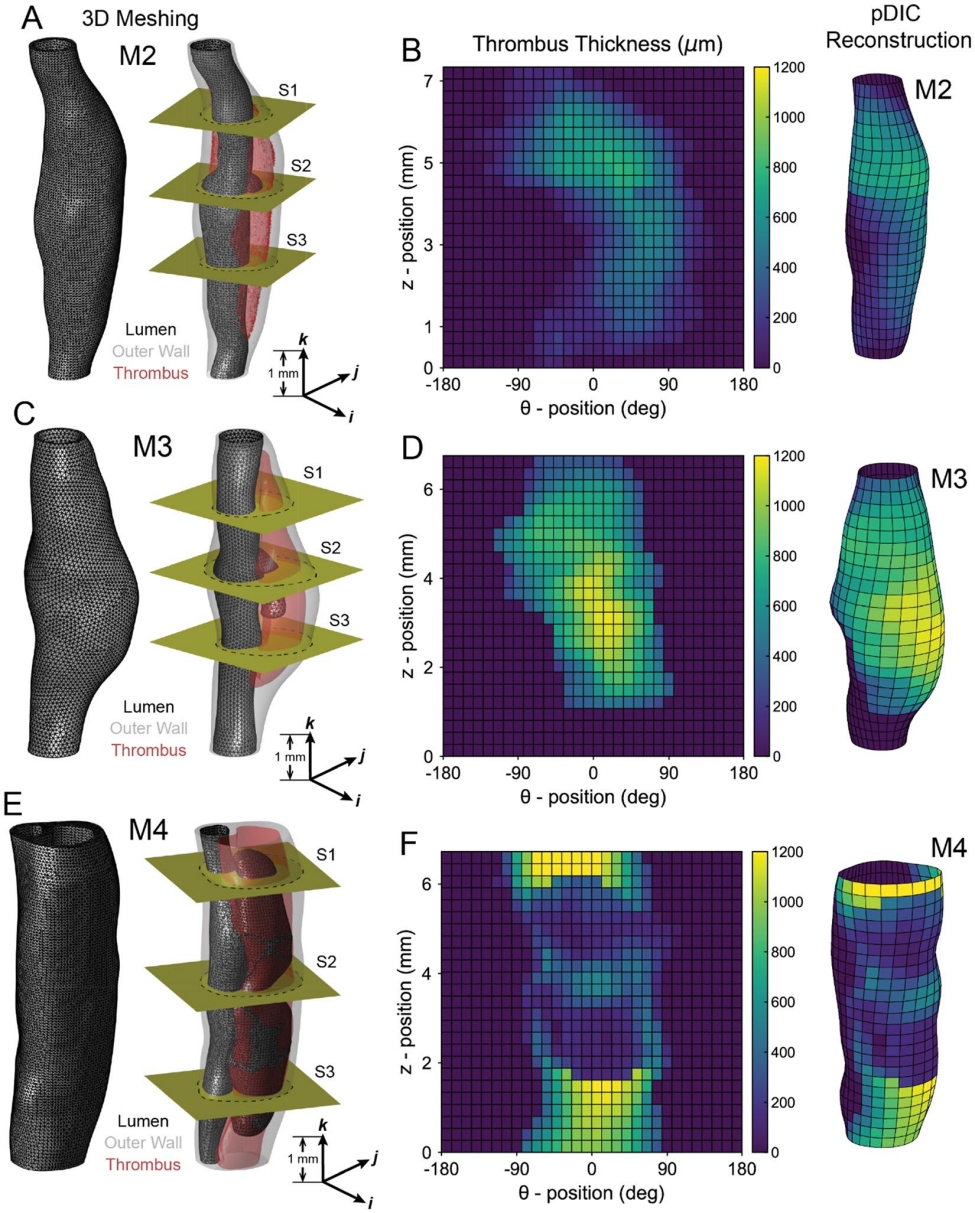
Geometric reconstruction of aortic dissection samples. Three-dimensional (3D) reconstructions of dissected samples (**A**) M2, (**C**) M3, and (**E**) M4 based on co-registered images from *in vivo* 3D-ultrasound and *in vitro* optical coherence tomography. Left: tetrahedral meshes appear as triangles on the adventitial and luminal surfaces. Right: visualization of internal luminal (black) and thrombus (red) surfaces in the three lesions, with the outer adventitial layer shown in transparent gray. Note that the false lumen was confined to small regions in M2 and M3 but extended almost the whole length of the sample in M4. Three cross-sections (denoted S1, S2, and S3) are shown (yellow cutting planes) for each sample. Quantitative analysis of samples (**B**) M2, (**D**) M3, and (**F**) M4 reveal spatial variations in the shape and size of intramural thrombus between samples. Samples are oriented such that regions with the thickest thrombus are oriented at 0°.

Using a prior co-registration between ultrasound and OCT images, high resolution finite element meshes of the true and false lumens were generated by manual segmentation^16^. Briefly, complete volumetric meshes along with surface meshes of the inner lumen boundary (black), outer adventitial boundary (gray) and thrombus boundary (red) were segmented for samples M2, M3, and M4 (Fig. 2A,C,E). Note the increasing thrombus volume and false lumen complexity from M2 to M4; false lumen boundaries are indicated in the black luminal boundary mesh. Using surface meshes for the lumen and thrombus boundaries, a full-field thrombus thickness was computed for each sample and overlaid onto the reconstructed pDIC geometry (Fig. 2B,D,F). In this parametric view, spatial variations in thrombus and false lumen thickness can be easily observed. For each sample in Fig. 2A,C,E, yellow slices (denoted S1, S2, and S3) represent axial locations that will be used for local comparisons between quantitative histological analysis and material property identification.

### Verification of OCT-DVC measurements

Surface deformations on the outer wall of dissected samples in response to extension-distension testing were measured by tracking motions of a speckle pattern using a custom pDIC technique^5,23^. Intramural deformations throughout the wall and thrombus were measured using OCT-based DVC^24,25^. These two sets of deformation measurements were then registered and combined in order to reconstruct the 3D reference geometry and compute the 3D finite strain fields throughout the specimen.

As this was the first study using OCT-DVC on murine arteries during extension-distension testing, we first performed multiple preliminary verifications of the technique. In a simple rigid body translation experiment of a pressurized SAA sample, we observed a good agreement between manually assigned translations and OCT-DVC inferred displacements in both the direction of the translation and the transverse direction (Fig. S3). This preliminary study confirmed the general accuracy of the OCT-DVC approach for measurement in mouse arteries and served as a validation of the set of parameters assigned in the global image processing and volume correlation algorithm. Note that the rigid body motion experiment was more a validation of the correct calibration between OCT and DVC than a validation of the measurement technique itself. Toward this end, we have previously applied OCT and DVC to arteries and observed very good performance of the technique^24,25^.

We also estimated standard deviations of the DVC-inferred displacements from the OCT images, which ranged from 10 to 15 µm. Note that this uncertainty is only for displacements in planes perpendicular to the axis of the vessel. Uncertainty could be one order of magnitude higher along the axis of the sample due to the larger voxel size in the axial direction. Indeed, only 100 axial cross-sections were scanned during OCT acquisition in order to minimize the duration of experiments (less than 12 hours) and reduce the amount of time that samples were maintained in physiologic saline solution outside of the body, noting that tissue degradation may begin to occur after 48 hours. Accordingly, the axial component of displacement *w* could not be accurately obtained and only the in-plane (*u*, *v*) displacement field was used from the OCT-DVC technique.

**Figure 3:**
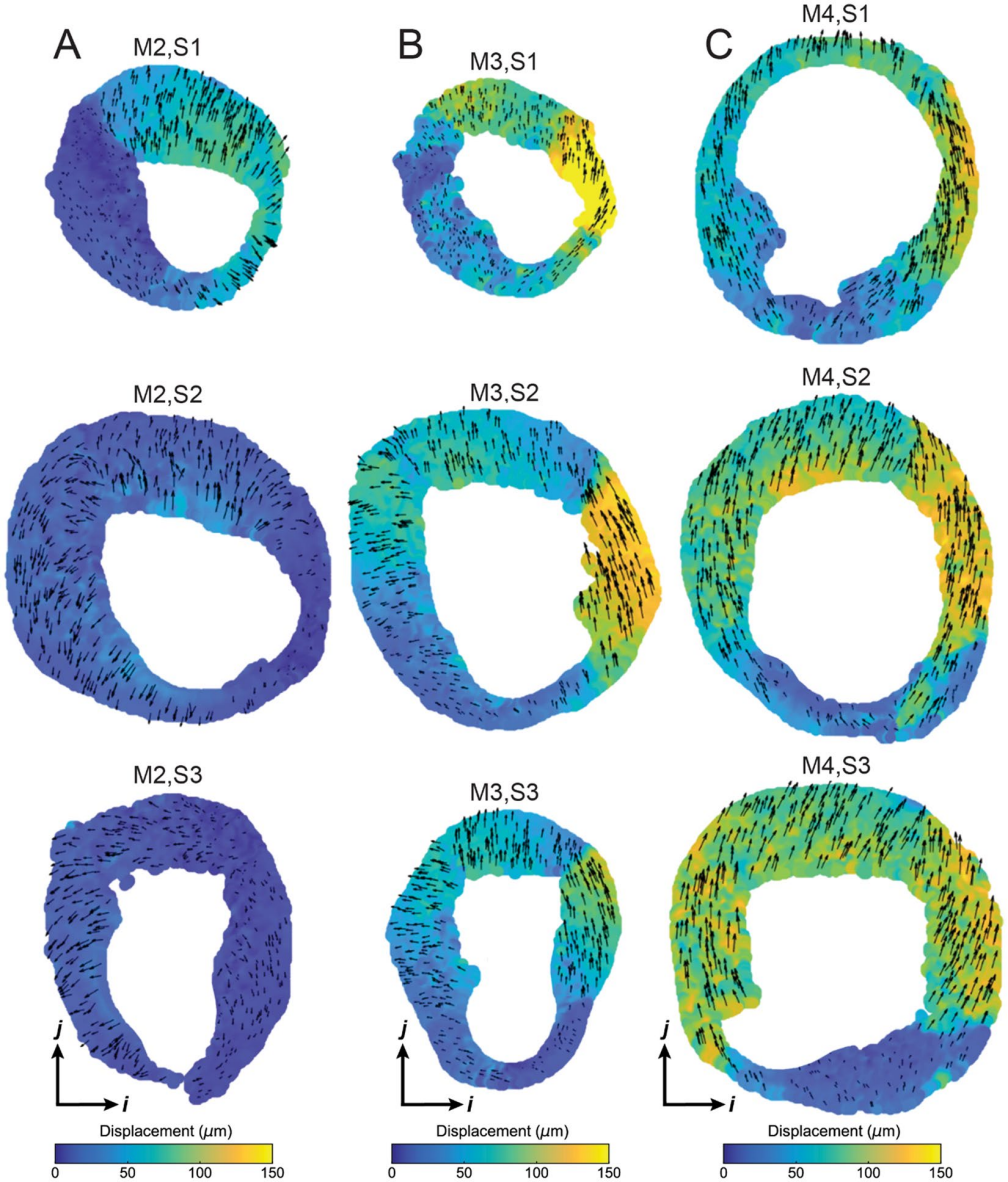
Intramural displacement measured by OCT-DVC. Measurements of local displacement magnitude from dissected samples (**A**) M2, (**B**) M3, and (**C**) M4 computed from OCT-based digital volume correlation (DVC) at cross-sections S1, S2, and S3 (ordered from top-to-bottom; cf. Fig. 2). Displacement maps are shown at the same scale (in μm). Arrows represent the direction of the displacement at each position, and their length is scaled to the local displacement magnitude.

The magnitude of the (*u*, *v*) displacement fields obtained from the OCT-DVC technique are shown in Fig. 3 for each thrombosed specimen (M2, M3, and M4) at multiple axial locations (S1, S2, S3; cf. Fig. 2A,C,E). These deformation measurements highlight both intra-sample spatial heterogeneities – with lower displacements often localized with the thrombus – and inter-sample variations – with sample M2 experiencing smaller deformations than both M3 and M4.

**Figure 4:**
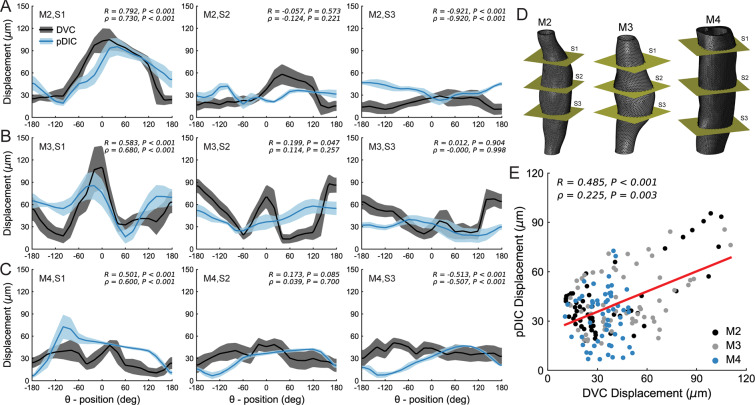
Comparison between pDIC and OCT-DVC displacements. Comparison of radial displacements at the surface of dissected samples (**A**) M2, (**B**) M3, and (**C**) M4 computed from volumetric-based OCT-DVC (black) or surface-based pDIC (blue). Representative results are shown at (**D**) cross-sections S1, S2, and S3 (left-to-right); note the variation in similarity between techniques. (**E**) Linear correlation analysis of all measurements reveals a reasonable relationship between measurement techniques (red line). Correlation coefficients and the corresponding *P*-values are included for each plot.

For each tested specimen (M2, M3, and M4), we also compared displacements on the external surface of the vessels measured by OCT-DVC to those measured by pDIC. Though differences were observed at several locations, there was an overall good agreement in terms of correlation coefficient values between the displacement measurements for each sample. Figure 4 shows representative comparisons between measurement techniques as a function of circumferential position for specimens M2, M3 and M4 (Fig. 4A–C) at three axial locations (again denoted S1, S2, S3; Fig. 4D). Though there were variations on an individual basis, comparison between OCT-DVC and pDIC displacements across all samples and locations showed a reasonable degree of correlation (*R* = 0.485, *P* < 0.001; Fig. 4E) suggesting that the two methods can capture similar displacement magnitudes, on average. Note that measurement differences between techniques need not be due to uncertainties in the OCT-DVC calculations. Indeed, the OCT-DVC and pDIC data were acquired separately – with the specimens mounted/removed between tests^22^ – and required co-registration which may have induced small spatial discrepancies in deformation measurements.

### Full-field reconstructions of mechanical properties in the aortic dissections

**Figure 5:**
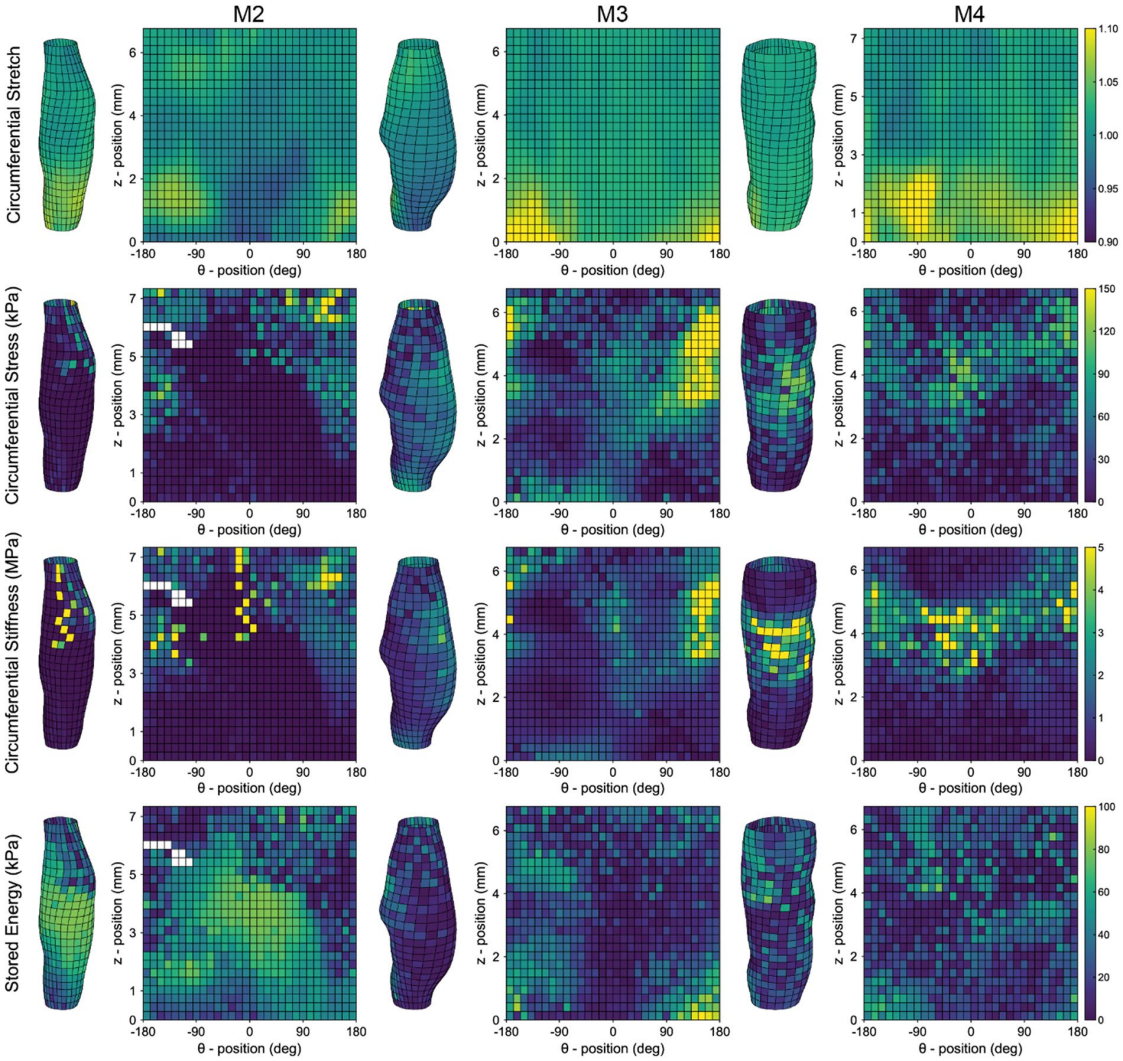
Spatial variations in representative computed mechanical metrics. Distribution maps show four key mechanical metrics computed at the surface of dissected samples M2 (first column), M3 (second column), and M4 (third column). Distributions of circumferential stretch (first row), circumferential Cauchy stress (second row, in kPa), linearized circumferential stiffness (third row, in MPa), and elastic stored energy (fourth row, in kPa) are shown. Values in the first three rows are shown at a distending pressure of 140 mmHg and stored energy is shown at a pressure of 80 mmHg; all measurements are shown at the *in vivo* axial stretch *λ*^iv^. Note the spatial variations within each identified distributions as well as the differences between samples.

Distributions of four key material metrics computed directly from the pDIC data – circumferential stretch (first row), circumferential Cauchy stress (second row), circumferential linearized material stiffness (third row), and stored energy (fourth row) – are shown in Fig. 5. For each tested specimen (M2, M3, and M4), data are shown at a common distending pressure of 140 mmHg and the *in vivo* axial stretch *λ*^iv^, recalling that the mice were hypertensive due to the AngII infusion. For the purpose of visualization, data were rotated so the maximum thrombus thickness is oriented at 0° (see Fig. 2B,D,F). Although these metrics are shown on the outer surface of the vessel, they can be computed at any radial position as the combination of OCT-DVC and pDIC permits reconstructions of 3D displacements and stretches at any position across each lesion. For example, Fig. S4 shows the circumferential stretch plotted on both the outer (adventitial) and inner (luminal) surfaces of the lesions of mice M2, M3 and M4; further, all reconstructed metrics from the non-dissected sample M1 are shown in Fig. S1. In the dissected samples M2, M3 and M4, the largest circumferential stretch occurs at distal ends (below a z-position of 2 mm) in the most distensible regions of the vessel, which are away from the dissection (out of the lesion and regions of intramural thrombus) and on the opposite side of the vessel from the false lumen.

Consistent with our prior pDIC studies^5,22^, the *in vivo* reference configuration was defined at 80 mmHg, hence the relative circumferential stretches at 140 mmHg are small (<1.15). The small circumferential stretches indicate a general stiffening of the wall due to *in vivo* hypertensive loading as well as remodeling in response to the dissection that had occurred ~7 days prior to vessel removal. Note that we observed a trend toward lower circumferential stretches in regions containing intramural thrombus (Fig. S5). Given the nonlinear (near exponential) mechanical behavior of the aorta, wall stress tends to reflect local values of wall stiffness^22^. In these lesions, some regions exhibited both a high stiffness and a high stress at 140 mmHg, as, for example, in sample M3 on the non-dissected side near the proximal end (i.e., at θ: 90°–180° and z: 3–6 mm). Yet, other regions exhibited a large stiffness while the associated circumferential stress remained low, as seen in both M2 and M4 around 0°. These findings may suggest the potential for localized changes in the underlying aortic wall composition. Finally, Fig. 5 also shows surface distributions of strain energy density at 80 mmHg and the *in vivo* axial stretch ${\lambda }^{{\rm{iv}}}$ for the same samples. Again, note the marked regional differences with low values tending to localize near regions containing thrombus, particularly in M3 and M4.

**Figure 6:**
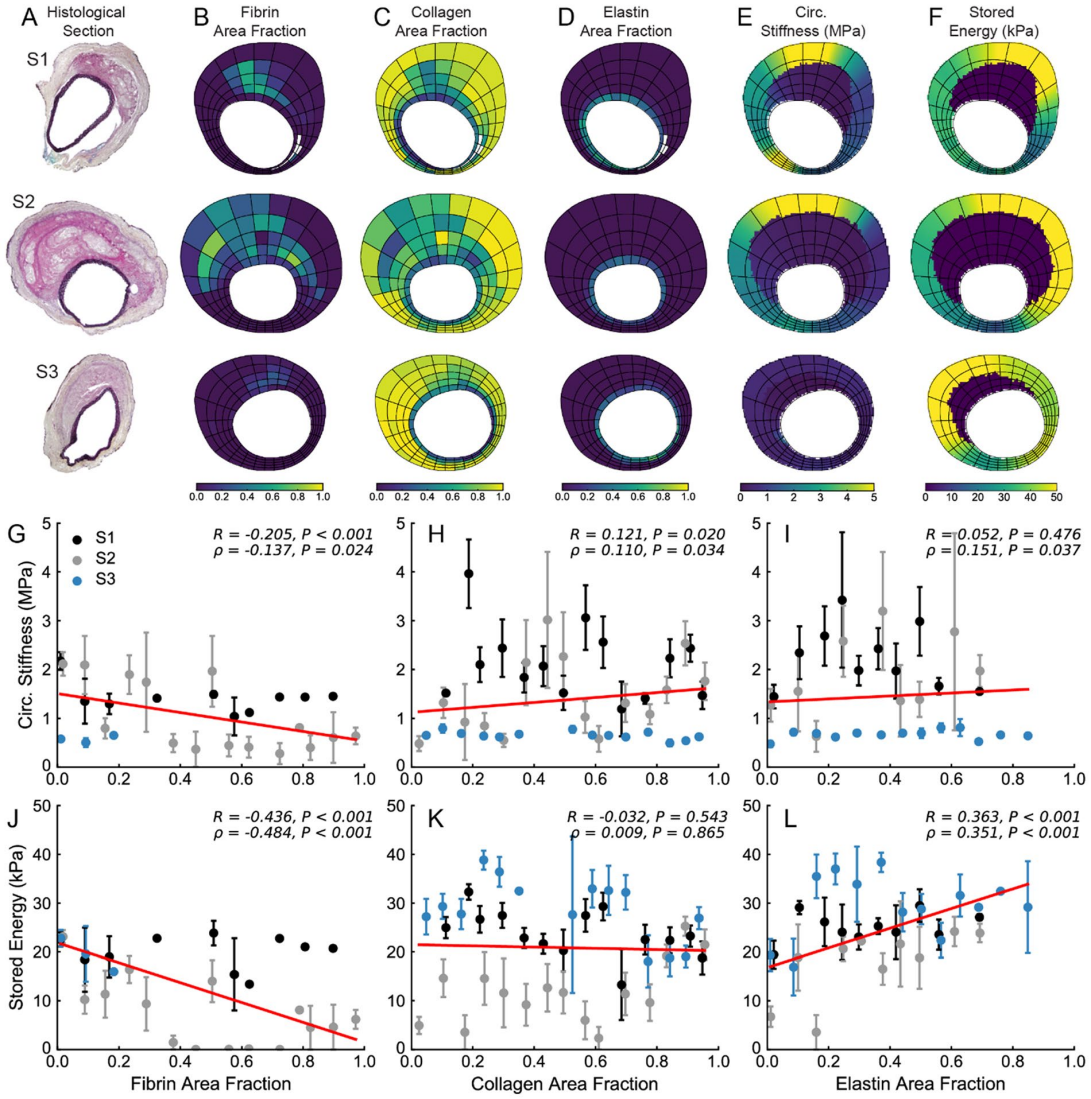
Local relationship between microstructure and material properties of M2. (**A**) At multiple histological locations (denoted S1, S2, and S3), quantitative histological analysis was performed in order to identify regional distributions of (**B**) fibrin, (**C**) collagen, and (**D**) elastin area fractions. Local values of (**E**) circumferential stiffness and (**F**) stored energy were also identified. Using the same spatial discretization, correlation analysis between local material properties and microstructure was performed. Circumferential stiffness and stored energy were plotted as a function of (**G**,**J**) fibrin, (**H,K**) collagen, and (**I,L**) elastin area fractions. Linear correlations are shown in red; correlation coefficients and the corresponding *P*-values are included for each plot.

**Figure 7:**
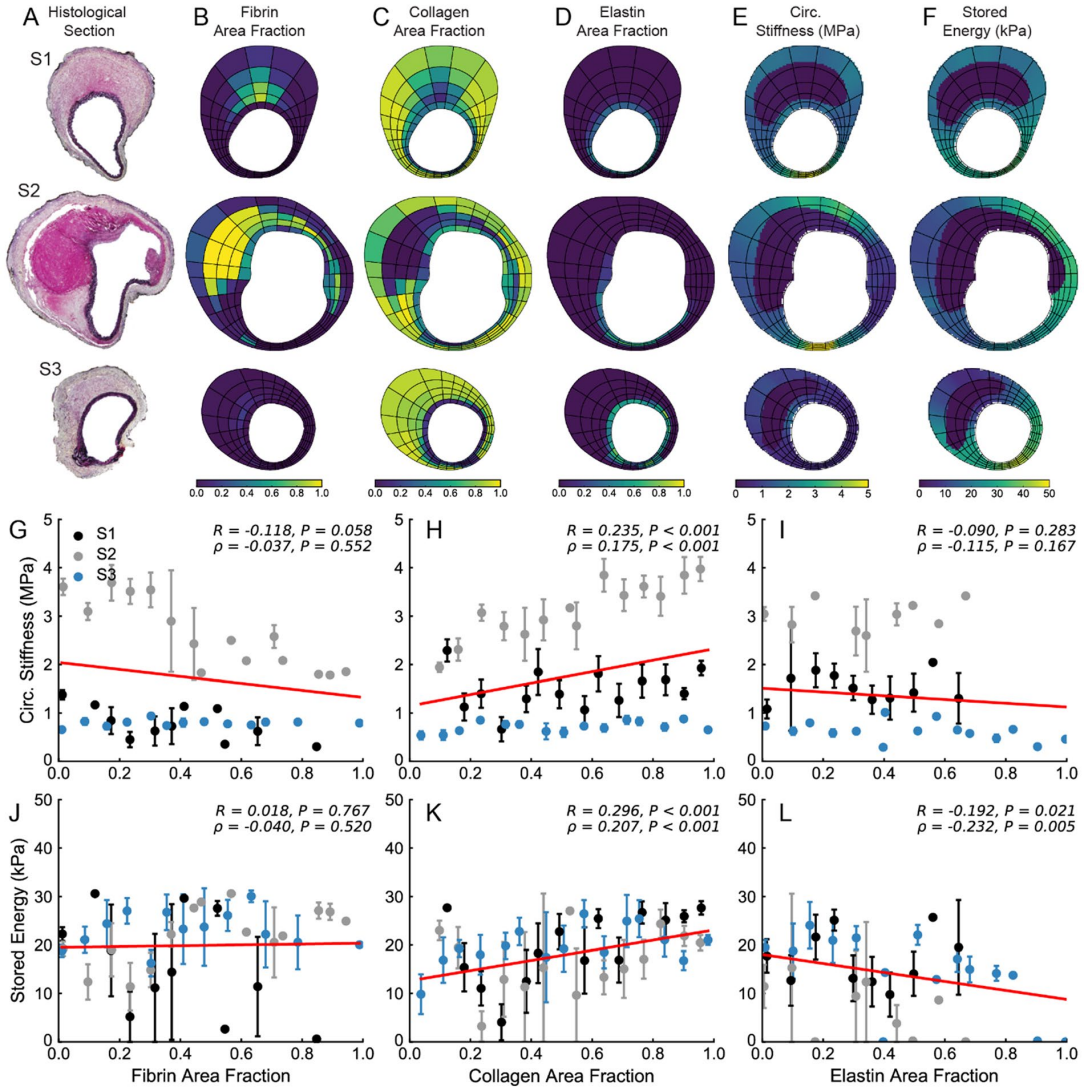
Local relationship between microstructure and material properties of M3. (**A**) At multiple histological locations (denoted S1, S2, and S3), quantitative histological analysis was performed in order to identify regional distributions of (**B**) fibrin, (**C**) collagen, and (**D**) elastin area fractions. Local values of (**E**) circumferential stiffness and (**F**) stored energy were also identified. Using the same spatial discretization, correlation analysis between local material properties and microstructure was performed. Circumferential stiffness and stored energy were plotted as a function of (**G,J**) fibrin, (**H,K**) collagen, and (**I,L**) elastin area fractions. Linear correlations are shown in red; correlation coefficients and the corresponding *P*-values are included for each plot.

**Figure 8:**
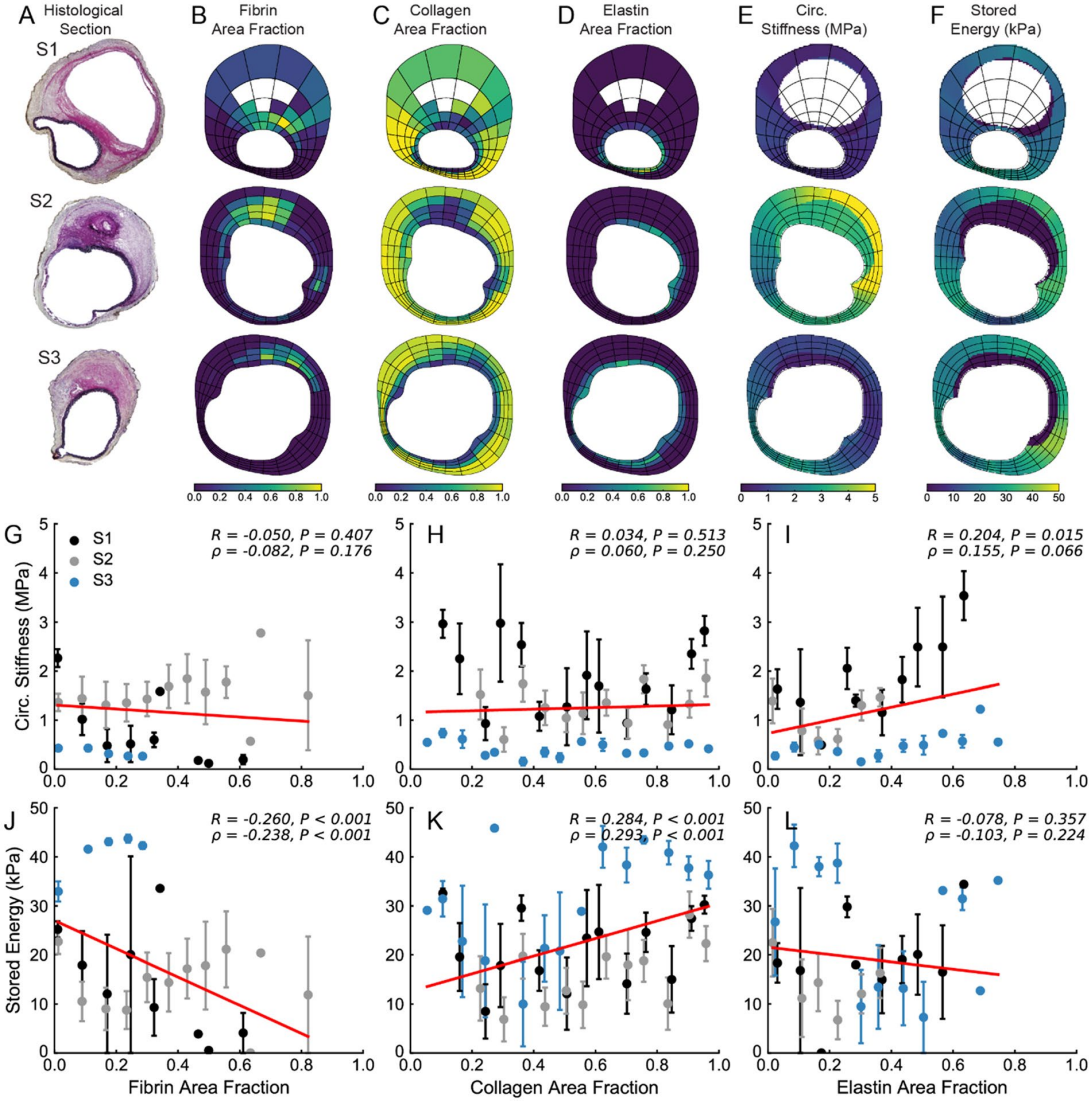
Local relationship between microstructure and material properties of M4. (**A**) At multiple histological locations (denoted S1, S2, and S3), quantitative histological analysis was performed in order to identify regional distributions of (**B**) fibrin, (**C**) collagen, and (**D**) elastin area fractions. Local values of (**E**) circumferential stiffness and (**F**) stored energy were also identified. Using the same spatial discretization, correlation analysis between local material properties and microstructure was performed. Circumferential stiffness and stored energy were plotted as a function of (**G,J**) fibrin, (**H,K**) collagen, and (**I,L**) elastin area fractions. Linear correlations are shown in red; correlation coefficients and the corresponding *P*-values are included for each plot.

All pointwise material property values were computed using the local best-fit material parameters from a four-fiber family hyperelastic constitutive model; parameters were identified using the proposed inverse method and the inferred full-field 3D strains (see Materials and Methods). Material properties were also identified using data combined from the OCT-DVC and pDIC measurements. Using this combined approach, local material properties and microstructural organization were quantified at cross-sections near those imaged for histology following registration to the OCT dataset based on thickness similarity. In Figs. 6–8, the processed histology sections (A) are shown along with discretized maps (containing 25 circumferential partitions and 5 radial partitions) showing local fibrin (B), collagen (C), and elastin (D) area fractions and the corresponding values of circumferential stiffness (E) and stored energy (F). Comparisons between M2 (Fig. 6), M3 (Fig. 7), and M4 (Fig. 8) revealed differences in properties between sections within a sample – consistent with a high degree of spatial heterogeneity – as well as differences across samples – consistent with varying degrees of thrombus burden.

In order to establish relationships between wall structure and properties, a linear correlation analysis was performed between stiffness, stored energy, and constituent area fractions (Fig. 6–8G–L). We observed overall trends toward decreased stored energy with increasing fibrin area fraction (J) and increased circumferential stiffness with increasing collagen area fraction (H); little effect was seen with elastin area fraction though a positive correlation with stored energy was observed for M2 (Fig. 6L). That local relationships between mechanics and microstructure may exist for one sample but not for another further highlights the inter-sample variation. As expected, the thrombus was significantly more compliant than the wall as shown by lower stiffness values (E), although some localized regions of thrombus in specimen M4 had a stiffness that approached that of the wall. These regions appeared to correspond to those wherein fibrin was remodeled into collagen. We also observed locations in all three specimens where the wall exhibited hotspots of circumferential stiffness reaching as high as ~4 MPa, which is ~2.5-fold greater than the baseline value of about 1.5 MPa for the SAA in control animals tested at normotensive pressures^5^.

It is important to note that stored energy was significantly lower in the dissected regions where the media was broken, and the stress was borne largely by the remnant adventitia. Overall, each lesion manifested uniquely heterogeneous distributions of properties – namely circumferential stiffness and stored energy – and wall composition that appeared to vary circumferentially, axially, and in many cases radially through the thick wall.

### Relationships between mechanical properties and wall composition

**Figure 9:**
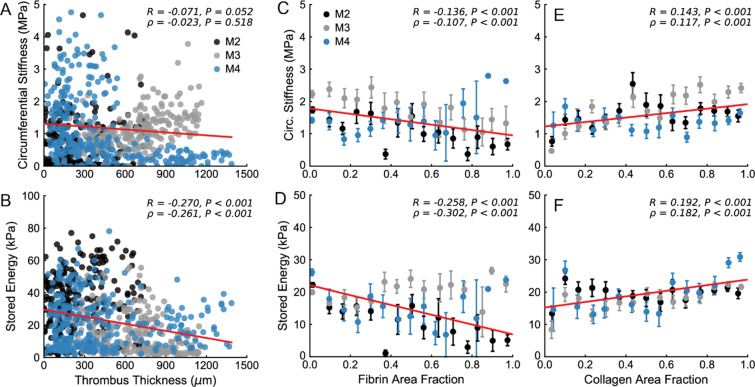
Global correlations between microstructure and material properties of aortic dissection samples. Comparisons between identified values of (**A**) circumferential stiffness and (**B**) stored energy and thrombus thickness (from Fig. 2) reveal slight decreasing trends across samples. Local trends in circumferential stiffness and stored energy as a function of (**C,D**) fibrin and (**E**,**F**) collagen area fraction reveal similar trends. Note the similarities between global and local analyses of intramural thrombus/fibrin and the slight inverse relationship between the impact of fibrin and collagen on material properties. Linear correlations are shown in red; correlation coefficients and the corresponding *P*-values are included for each plot.

Looking at global trends in the effect of intramural thrombus (from Fig. 2) on the identified mechanical properties of the dissected aortic wall, we observed little effect on circumferential stiffness (Fig. 9A) but a trend toward decreased stored energy with increasing thrombus thickness (Fig. 9B). Local relationships between computed values of circumferential stiffness, stored energy and the area fractions of fibrin, collagen, and elastin are shown for each sample in Figs. 6–8. On an individual basis, there was generally a decrease in circumferential stiffness and stored energy with increasing fractions of fibrin (for instance in Figs. 6G, 7G and 8G). However, partly because the samples and cross-sections differ greatly in geometry, composition, and proximity to different types of thrombus, and partly because we did not quantify constituent orientation, undulation, or cross-linking, less significant trends arose when considering all local measurements of mechanical properties and histology, as revealed by lower correlation coefficient values in Fig. 9C,D. That said, we did observe slight negative correlations between fibrin area fraction and both circumferential stiffness and stored energy (Fig. 9C,D) – similar to the global trends with thrombus thickness (Fig. 9A,B). We also observed slight positive correlations between collagen area fraction and both circumferential stiffness and stored energy (Fig. 9E,F), suggesting a mechanical difference between fibrin-rich and collagen-rich portions of the dissected wall and highlighting the potential for changes in properties over the course of thrombus maturation and fibrin turnover. No trends were observed with elastin area fraction (Fig. S6), further highlighting that the overall mechanical response is likely dominated by the turnover of fibrin and collagen.

Further aspects should probably be considered to establish better links between mechanical properties and histology. In particular, the fraction of elastin and collagen can only be linked to material properties if one knows the structure and state of elastin and collagen fibers: damage and disruption of fibers decrease stiffness whereas crosslinks induce an increase in stiffness, for similar mass fractions. Although it is expected that a normal blood vessel would adapt to return wall stress toward its homeostatic target – for instance by depositing collagen fibers in cases of increased pressures – the three dissected lesions likely have compromised homeostasis and are at various stages of remodeling and tissue repair. This, too, may explain the lack of strong correlation between local microstructure and mechanical properties, thus emphasizing the importance of quantifying functional metrics and not just composition.

## Discussion

Aortic dissection can be life-threatening, yet its location tends to dictate the current standard of clinical care. Dissection of the ascending aorta (Stanford type A) often leads to death within days if untreated^26^; urgent or emergency surgery typically involves replacing the affected section with a synthetic graft. Dissection of the descending aorta (Stanford type B) can also be fatal but is often managed medically over long periods by controlling blood pressure to reduce wall stress. It is also thought that type B dissections that exhibit either a patent or a fully thrombosed false lumen have a better prognosis than those that exhibit a partially thrombosed false lumen^27^. A partial false lumen may allow the thrombus to be replenished with blood-borne cells and proteins that may drive a chronic inflammatory response that can weaken the wall; a fully thrombosed false lumen, on the other hand, may heal, leading to the replacement of fibrin with collagen fibers^4^, which have greater stiffness and strength. Computational biomechanical models are needed to understand both the role of blood flow in the formation of thrombus within a false lumen^18^ and the potential maturation of thrombus *in vivo*^28^. The former is aided greatly by detailed studies of the hemodynamics, as reported for human dissections^29^ and animal models^16^. There is, in addition, a pressing need for fluid-solid interaction studies, and similarly biomechanical analyses, of the wall itself. This is particularly important since the primary concern is rupture and extravasation of blood due to transmural failure of the aorta when wall stress exceeds wall strength.

Because of the complex geometry and highly heterogeneous composition and properties associated with aortic dissection, biomechanical studies of the dissecting or dissected wall have necessarily been limited. Most experimental studies focused on the mechanics of aortic dissection have employed standard peel-type tests on aortas from humans or large animals to evaluate the local transmural strength of the wall^30,31^. Such studies provide invaluable information regarding the dissection process, including that which is needed to simulate intramural delaminations that could lead to the formation of a false lumen or rupture of the wall^32,33^. Other computational simulations have focused on the potential role of pooled proteoglycans in initiating a dissection^34^, a hypothesis supported by recent human data^35^. Yet, longitudinal data remain limited, which motivates the use of small animal models when studying such mechanisms.

Although aortic lesions that arise in the AngII-infused *ApoE*^−/−^ mouse do not phenocopy human dissections in many regards – they occur in the abdominal, not thoracic, aorta near major branch sites, they occur at the interface between the media and adventitia rather than within the media, they do not result from a connective tissue disorder, and they do not appear to involve focal accumulations of glycosaminoglycans – they nevertheless represent an important model for study. Recent studies have used this model to gain insight into various molecular components of aortic dissection, including lymphocyte adaptor protein deficiency^36^, smooth muscle α-actin deficiency^37^, ciprofloxacin treatment^38^, and transforming growth factor-β–SMAD3 signaling^39,40^.

Using this model, we also recently identified a brief period during early AngII infusion wherein matrix degradation appears to outpace deposition only in the SAA, perhaps increasing the vulnerability of this region to the increased wall stress that comes from the early elevation in blood pressure^2^. This observation was based, however, on histomechanical data that resulted from standard biaxial testing of non-dissected samples, which yields bulk not local properties in the absence of thrombus. There was a need for more detailed analyses of the wall mechanics following aortic dissection, which motivated this multimodality-based study. The current paper appears to be the first to document the highly heterogeneous wall properties and wall stresses throughout the dissected region of the SAA in the AngII-infused *ApoE*^−/−^ mouse, though pDIC has been used with this model previously^41^. Importantly, the present results can be compared with prior pDIC-based results for a normal SAA in the *ApoE*^−/−^ mouse wherein nonuniform distributions of wall properties arise primarily near the major branches (celiac, superior mesenteric, etc.) within this region of the aorta^5^. Significant differences between dorsal and ventral locations were also observed in the normal SAA, which may result from differential perivascular support. Not surprisingly, the present results confirm that each lesion – which span a broad range of false lumen geometries and blood flow^16^ – is unique biomechanically, hence suggesting that it will be difficult to identify the biomechanical phenotype based on *in vivo* imaging alone.

Since the pioneering work of Fillinger *et al*.^42^, many studies have highlighted the potential of finite element based stress analyses for predicting rupture risk in aortic aneurysms^43–47^ and dissections^48,49^. For the latter, recent studies suggest possible correlations between positions of peak wall stresses and locations of dissection initiation^50,51^, but most of these models assume uniformly distributed material properties. In contrast, our results demonstrate the existence of dramatic regional variations in material properties that can vary axially, circumferentially, and even radially, within these complex lesions. Such spatial variations are likely a result of immuno-mechano-biological adaptations and post-dissection damage, remodeling, and healing. Prior studies have proposed strain-based, rather than stress-based, rupture criteria to predict the risk of aortic dissections^52–54^. Yet, locations of calculated hotspots of maximal principal strain are also likely affected by the assumed distribution of material properties. Indeed, non-uniform axial stretch, non-uniform wall thickness, and the presence of minor subcostal arteries were all shown to significantly alter rupture criteria and predictions of risk^55^. Even though experimental data describing the distribution of material properties, or other geometric and material features, are often not available in clinical practice, one should be aware of the important implications that model simplifications might have on the final simulated result. Toward this end, the use of virtual histology and advanced imaging techniques^13,56^ could permit one to inform finite element models with relevant distributions of material properties, provided that the histological composition can be related to appropriate mechanical parameters.

Numerous constitutive models in vascular mechanics are motivated by the tissue microstructure^57,58^. Correlations between local features of the microstructure and material behavior could thus be helpful in guiding the constitutive formulations that are needed to perform detailed stress analyses for the purposes of predicting susceptibility to failure. Such features include local fractions of structural proteins such as elastin and collagen, but also their local organization into fibrous networks. Recently, the combination of mechanical tests with second harmonic generation / two-photon fluorescence microscopy^59–61^ has enhanced our ability to track collagen and elastic fibers under different mechanical loads and how these fibers contribute to the local mechanical properties. As we were able to register cross-sectional histological images with our mechanical reconstructions, we were able to evaluate potential correlations between relevant mechanical quantities, such as the linearized circumferential stiffness or the stored energy density, and local fractions of collagen, elastin, or fibrin. Of particular interest was the potential mechanical implication of fibrin being replaced by collagen in the intramural thrombus. The present results suggest that such relationships exist locally (Fig. 9C–F), however local fiber orientations and undulations must also be quantified in order to strengthen the identified trends, even when comparing dissimilar materials like fibrin and collagen. Combining our inverse technique with multiphoton microscopy appears to be a promising avenue for future study as we seek to identify links between macroscopic mechanical properties and the underlying microstructural composition.

Regarding possible clinical translations, the extension of this methodology to *in vivo* data such as US and MRI, remains a major challenge^54,62^. Our approach reported in this paper is better suited for research in vascular biology and mechanics where one wants to assess the effects of surgical, genetic, or pharmacological treatments on arterial mechanobiology using animal models. That said, we sought to identify correlations between tissue microstructure and mechanical properties (Figs. 6–9) in order to gain a better understanding of tissue remodeling associated with aortic dissection while introducing a new methodology and mathematical framework. With future studies focused on characterizing such relationships over time and in various treatments, relationships between thrombus properties, disease progression, and stiffness can be further developed. Such general trends could then presumably be used to compare with clinical data in an effort to develop a better understanding of thrombus properties on disease progression in the context of aortic dissection.

We emphasize, nonetheless, that lessons learned from more complete *in vitro* data can often help inform *in vivo* assessments, particularly with regard to appropriate functional forms of constitutive relations, ranges of values of material parameters, and potential trends in spatiotemporal changes.

### Limitations

While new information can be gained via this OCT-DVC + pDIC based inverse characterization, there are limitations to this approach.

pDIC measurements were necessarily performed while the samples were placed within a conical mirror. Hence, the OCT and pDIC measurements were not simultaneous; technical challenges with this discrepancy included possible non-loading induced motions when moving the sample from one system to the other, difficulties in registering the different images, and extending the duration of experiments and time that samples were maintained in physiologic saline solutions outside of the body. The virtual fields based inverse methodology also required at least 6 hours for the complete identification of material properties per sample; however, this modest computational expense could be reduced by implementation of parallelization techniques.

The spatial resolution of the imaging modalities is also a crucial aspect. The OCT system used in this study had an in-plane (transverse) voxel size of 7 × 7 µm². Although this allowed for reconstruction of meshes containing small details of the true lumen, false lumen, and thrombus^16^, stiffness reconstructions could not reach such level of resolution due to the smoothing effects in the subsequent DVC analysis (linear approximation of Eq. 2). Moreover, the axial resolution, which is based upon sampling in the axial direction during data acquisition, was 65 µm, on average. The axial resolution is the main limitation of this approach and could have an impact on segmentation of the length of ruptured media. Nevertheless, in our samples, the dissected region was always at least 0.4 mm long, thus the spatial resolution along the axial direction was never critical.

Another limitation is related to side branches, which had to be ligated near the ostia in order to perform the pressurization experiments and make the external surface of the vessel amenable to pDIC measurements. In the field of view of the vertically mounted camera, long branches can create artifacts that result in regions on the vessel surface that cannot be reconstructed. Although we can reconstruct FE models with side branches, by ligating branches during the *in vitro* testing, the deformations of side branches could not be measured and therefore material properties could not be identified.

Moreover, the proposed methodology for complex thick-walled lesions such as dissected aneurysms required a very specific methodology combining OCT, DVC, pDIC and the virtual fields method. Together, this results in labor and time intensive post-processing procedures. However, the initial methodology^5^ for thin aortic samples (relying only on pDIC for strain measurements) was also labor intensive at that time and the procedure has been progressively automated which now permits its use for larger datasets to study thoracic aortic aneurysms. Similar progress is possible for the current methodology. For instance, automatic meshing and registration of imaging modalities, and fast OCT acquisition at specific cross sections combined with pDIC, would significantly reduce the effort.

The distribution of material properties across the thickness of the wall was also based on assumptions on image-informed morphology as we were not able to resolve the gradients of material properties across the thickness due to the light penetration depth of the OCT imaging system. The maximum penetration depth is ~2 mm, but the penetration depth needed to keep enough contrast in the signal is ~0.4 mm, which is smaller than the thrombus thickness in some dissected regions of the samples. To overcome the problem of complete penetration depth, we merged rotationally-symmetric images and neglected radial variations in material properties by assuming linear variations of displacements in the radial direction. This allowed us to extrapolate the displacements and strains across the whole thickness even in the region where the OCT could not penetrate to reach the lumen. Acquiring OCT images at intermediate loading steps and improving their resolution could probably address these issues, but this would dramatically lengthen the durations of the experiment.

## Conclusion

We submit that the present multimodality based approach to inverse characterization of murine arteries will enable increasingly detailed assessments of heterogeneous wall strains and stresses – but especially material properties – of complex vascular pathologies. Although illustrated for the AngII-infused *ApoE*^−/−^ mouse model, this approach can also be used to study myriad mouse models of aneurysms and dissections that have previously been examined via standard biaxial testing (e.g., type II transforming growth factor receptor disruption^63,64^ and smooth muscle myosin heavy chain mutation^65^ to name a few). Although no single mouse model phenocopies exactly a human disease, we submit that comparisons across multiple aortic regions or mouse models^1–3^ has tremendous potential to increase our understanding of disease progression. Further, the ability to develop local relationships between material properties and tissue microstructure^22^ can provide a better mechanical understanding of complex vascular pathologies and enhance the characterization of disease states from medical imaging alone. Eventually, additional extensions of the methodology and inverse identification will include taking advantage of the thousands of independent mechanical responses for each sample to set up statistical models through Bayesian inference^66^ or physics informed neural networks^67^.

## Materials and Methods

### Animal model

The four mice used in this study were the same as those used to perform complementary hemodynamic calculations^16^. For completeness, however, note that the Institutional Animal Care and Use Committee at Purdue University approved all animal procedures and all methods were carried out in accordance with relevant guidelines and regulations. Alzet mini-osmotic pumps were surgically implanted in adult male *ApoE*^−/−^ mice to deliver AngII at 1000 ng/kg/min for up to 28 days. The SAA was monitored every 48 hours post-operatively using ultrasound to detect the initiation of a dissection event. Of the seven mice implanted, one presented with an enlarged aortic wall without dissection (M1) and three developed dissecting aneurysms of varying severity (M2, M3, and M4); the other three mice did not dilate or dissect and were not studied further.

For each of the four study mice, we acquired axial, sagittal, and transaxial three-dimensional ultrasound (US; MS550D 22–55 MHz transducer, Vevo2100, FUJIFILM VisualSonics) datasets before surgery (baseline), on the day of diagnosis of SAA expansion (day 0), and at four times after diagnosis (days 1, 3, 5, and 7). The last US measurement was used to create computational domains for the hemodynamics^16^, as well as for the finite element model of the wall as used herein (Fig. 2). Below, we focus on quantification of the associated local mechanical properties of the dissected wall that was enabled by melding three complementary imaging modalities, two of which (pDIC and OCT) were used *in vitro* on the excised SAA.

### SAA preparation and biomechanical testing

Following euthanasia, we flushed the aorta with a standard physiological buffered solution (1 mL/min for 5 min) by transcardial perfusion through the left ventricle. We then excised the aorta en bloc, from the heart to the iliac bifurcation, paying special attention to preserve infrarenal and suprarenal branches, surrounding connective tissue, and the spine. Specimens were kept in ice-cold phosphate-buffered saline (PBS) until ready for *in vitro* testing, within 24 hours of death.

Each specimen was first mechanically tested using a custom biaxial device and a well-established *in vitro* testing protocol^20^. The SAA was isolated and prepared for testing by removing excess perivascular tissue and ligating all branches using single strands from braided 7–0 nylon suture. Then, the two extremities of the SAA were coupled securely to glass cannulae using 6-0 sutures and the mounted specimen was placed within a custom, computer-controlled biaxial testing system^68^ and subjected to cyclic pressurization from 0 to 140 mmHg at three different fixed axial stretches (the *in vivo* value, denoted *λ*^iv^, and ±5% of this value). The *in vivo* axial stretch was defined as the value that minimized variations in transducer measured axial load upon pressurization^68^. Note that the *λ*^iv^ determined with this technique was applied to the artery when it was mounted in the OCT imaging system. After aligning the OCT images with the US images (which were acquired *in vivo*), a very good overlap was obtained between the two images^16^, confirming that the *in vivo* axial stretch estimation was accurate. All tests were performed in Hanks Buffered Salt Solution (HBSS).

The whole experiment was carried out on the same day, when possible. Tissue was prepared for mechanical testing (1–3 hours), followed by OCT imaging (1–2 hours) and pDIC testing (3–5 hours). The total experimental protocol was typically performed in less than 10 hours, with the maximum time between OCT and pDIC data acquisition taking around 6–7 hours.

### pDIC measurements

Following standard biaxial testing, specimens were recannulated at the same locations on a single blunt-ended needle, oriented vertically, and placed within a custom panoramic-digital image correlation (pDIC) system^23^ to monitor full-field surface deformations at multiple states of pressurization (every 10 mmHg from 0 to 140 mmHg) and axial stretching (the same three used during computer-controlled biaxial testing). A set of ${{\rm{N}}}_{{\rm{pDIC}}}$ surface positions were defined, forming a grid across the adventitial surface of the artery. The displacement fields were calculated at these positions from the surface deformations at each applied pressure and axial stretch as described previously^5,22,23^.

### OCT measurements

Following data collection using pDIC, the recannulated SAA was also scanned with a commercial optical coherence tomographic (OCT) system (ThorLabs, Inc., NJ) to provide through-the-wall information with an in-plane spatial resolution of 7 μm for each cross-sectional image. OCT images were acquired at multiple states of pressurization (every 20 mmHg from 0 to 140 mmHg) and axial stretching (the same three used during biaxial testing and pDIC measurements). A complete 3D OCT image consists of 100 cross-sectional images. The distance between two cross-sectional images varied between 65 and 80 µm, depending on the length of the sample. Consequently, there is one order of magnitude difference between the in-plane cross-sectional spatial resolution and the axial spatial resolution for the OCT images. Note also that for each cross-section, four OCT images were acquired from four different angular views (−90°, 0°, 90° and 180°) because of the increased outer diameter of the sample. The set of images for each angular view were combined to obtain the final complete 3D OCT image. Registration between each angular view was achieved using 3DSlicer^22^.

### Mesh reconstruction

The 3D US datasets obtained *in vivo* at day 7 were co-registered with the OCT datasets obtained *in vitro* at 80 mmHg and the *in vivo* axial stretch, *λ*^iv^. Merging these two datasets provided detailed information on the geometry of each aortic lesion and the nearby vasculature (including both the true lumen and the enclosed false lumen present in dissected samples). The combined image resolution was high enough to reconstruct computational domains suitable for basic structural analyses of the dissected wall, with delineation of regions including the lumen, intramural thrombus, and the aortic wall (Fig. 2). Using semi-automatic segmentation algorithms, we extracted luminal, thrombus, and outer wall boundaries at several cross-sections along the SAA. The segmented boundaries were then lofted. The whole 3D luminal boundary is denoted S_in_ and the whole 3D outer wall boundary is denoted S_out_.

Finally, the luminal, thrombus, and outer wall boundaries were merged to form the requisite 3D domains aligned with the image datasets. The whole 3D computational domain encompassing the volume located between S_in_ and S_out_ is denoted Ω, where $\Omega ={\Omega }_{{\rm{w}}{\rm{a}}{\rm{l}}{\rm{l}}}\oplus {\Omega }_{{\rm{i}}{\rm{m}}{\rm{t}}}$, with Ω_imt_ the domain occupied by the intramural thrombus and Ω_wall_ the domain of the aortic wall. The complete Ω domain was discretized into N_Ω_ tetrahedral elements (N_Ω_~150,000), with ${{\rm{N}}}_{{\Omega }_{{\rm{wall}}}}$ tetrahedral elements in Ω_wall_ and ${{\rm{N}}}_{{\Omega }_{{\rm{imt}}}}$ tetrahedral elements in Ω_imt_. S_in_ and S_out_ comprised, respectively, N_in_ and N_out_ triangular facets (see Fig. 2).

### Digital volume correlation (DVC)

The OCT data were saved as 3D images in TIFF format and the TIFF virtual stack was imported using ImageJ software. The data were rescaled and converted to 8 bits for intensity level digitalization. The OCT data was exported as RAW image format and full-field displacements were computed using the DVC method^24^. Multipass schemes available in DaVis-DVC (LaVision) allowed us to achieve decreasing sub-volume sizes of 24-20-16-12-10-8 voxels, to find the displacement field ${\boldsymbol{u}}=(u,v,w)$, using two iterations each time except the last which used 10 iterations.

Like the pDIC method, the OCT-based DVC method was implemented for every combination of distending pressure and axial stretch to find the incremental displacement fields between consecutive states of pressurization (20 to 40 mmHg, 40 to 60 mmHg, and so on until 120 to 140 mmHg) as well as the different axial stretches (namely between *λ*^iv^ and $1.05{\lambda }^{{\rm{iv}}}$ and between *λ*^iv^ and $0.95{\lambda }^{{\rm{iv}}}$). Ultimately, all incremental displacement fields were combined to estimate the displacement field of each configuration with respect to a unique *in vivo* relevant reference configuration defined at 80 mmHg and the *λ*^iv^ axial stretch.

The resulting displacement fields were written as arrays at all positions (x, y, z), which are centered within each subvolume and yielded a 3D grid representing the whole SAA segment of interest. For every configuration, this represents 256 × 256 × 50 positions. A mask was defined to eliminate all non-essential positions outside of Ω from the analysis, and the displacement was estimated only at the remaining N_DVC_ positions. Additional details can be found in the Supplemental Methods.

### Co-registration of OCT-DVC and pDIC data

The OCT data that needed to be aligned with the pDIC data included the region of the SAA containing the dissection as well as the near distal and proximal abdominal aorta. As deformations induced by mounting and unmounting were negligible, and as the axis of the artery was already pre-aligned for both imaging techniques, the co-registration only required determination of: (1) the rotation about the arterial axis and (2) the translation along this axis. All outer wall points from pDIC reconstruction and OCT segmentation were selected, and the unknown rotation and translation were found by minimizing the distance between each set of points using an in-house MATLAB routine. In this case, the degree of overlap following registration is a good criterion as the arteries tested in this study were highly asymmetric (with the dissection located on one side) and exhibited significant diameter variations. Due to the complex morphology there was no ambiguity with the overlapping criterion to judge about the success of the co-registration.

Use of OCT-DVC to measure displacement fields across blood vessels is recent^24,69–71^. Our study is the first to use it on the murine aorta, hence we performed a supplementary experiment to evaluate the accuracy of this technique for such small samples (Fig. S3). This validation consisted of applying incremental translations to a segment of SAA (i.e., a biaxially cannulated segment placed on a micro-translation stage and translated rigidly) and scanning the sample with the OCT device after each increment. Data were processed using our DVC algorithm and compared to the imposed translations in both the direction of the translation and in the transverse direction. We also compared computed displacements inferred from the OCT-DVC data to those surface values inferred using pDIC, which can be considered as a validated reference technique^23^.

### 3D strain reconstruction

The displacement vector is denoted ${\boldsymbol{u}}({\boldsymbol{X}},t)$ for any material point, with coordinates ${\boldsymbol{X}}=X{\boldsymbol{i}}+Y{\boldsymbol{j}}+Z{\boldsymbol{k}}$ in the reference configuration at time *t* (which denotes a quasi-statically achieved equilibrium configuration, as, for example, a deformed configuration corresponding to one combination of axial stretches and applied pressures). The reference configuration ${\Omega }_{0}$ is set at 80 mmHg and at the *λ*^iv^ axial stretch. For convenience, the position of material points was also defined with cylindrical coordinates:

1 $${\boldsymbol{X}}=R\,\cos (\Theta ){\boldsymbol{i}}+R\,\sin (\Theta ){\boldsymbol{j}}+Z{\boldsymbol{k}}.$$

For pDIC measurements, we measure displacements at ${{\rm{N}}}_{{\rm{pDIC}}}$ positions forming a grid across the outer surface ${{\rm{S}}}_{{\rm{out}}}$ defined such as: ${Z}_{{\rm{d}}{\rm{o}}{\rm{w}}{\rm{n}}}\le Z\le {Z}_{{\rm{u}}{\rm{p}}}$, $-\pi \le \varTheta  < \pi$ and $R={R}_{{\rm{o}}{\rm{u}}{\rm{t}}}(\varTheta ,Z)$. For DVC measurements, we measured displacements at ${{\rm{N}}}_{{\rm{DVC}}}$ positions forming a 3D grid across the domain ${\Omega }_{0}$ defined such as: ${Z}_{{\rm{down}}}\le Z\le {Z}_{{\rm{up}}}$, $-\pi \le \Theta \le \pi$ and ${R}_{{\rm{in}}}(\Theta ,Z)\le R\le {R}_{{\rm{out}}}(\Theta ,Z)$. In order to combine both measures into a single displacement at every position across ${\Omega }_{0}$, we assumed that transverse displacement components $\tilde{{\rm{u}}}$ satisfied the following distribution:

2 $$\begin{array}{ccc}\mathop{u}\limits^{ \sim }(R,\varTheta ,Z,t) & = & \mathop{\sum }\limits_{{\rm{n}}=1}^{{{\rm{N}}}_{{\rm{p}}{\rm{D}}{\rm{I}}{\rm{C}}}}\zeta (\varTheta -{\varTheta }_{{\rm{n}}},Z-{Z}_{{\rm{n}}})[\frac{R-{R}_{{\rm{i}}{\rm{n}}}({\varTheta }_{{\rm{n}}},{Z}_{{\rm{n}}})}{{R}_{{\rm{o}}{\rm{u}}{\rm{t}}}({\varTheta }_{{\rm{n}}},{Z}_{{\rm{n}}})-{R}_{{\rm{i}}{\rm{n}}}({\varTheta }_{{\rm{n}}},{Z}_{{\rm{n}}})}{u}_{{\rm{p}}{\rm{D}}{\rm{I}}{\rm{C}}}({\varTheta }_{{\rm{n}}},{Z}_{{\rm{n}}},t)\\  &  & +\frac{{R}_{{\rm{o}}{\rm{u}}{\rm{t}}}({\varTheta }_{{\rm{n}}},{Z}_{{\rm{n}}})-R}{{R}_{{\rm{o}}{\rm{u}}{\rm{t}}}({\varTheta }_{{\rm{n}}},{Z}_{{\rm{n}}})-{R}_{{\rm{i}}{\rm{n}}}({\varTheta }_{{\rm{n}}},{Z}_{{\rm{n}}})}{u}_{{\rm{i}}{\rm{n}}}({\varTheta }_{{\rm{n}}},{Z}_{{\rm{n}}},t)]\end{array}$$

where ${u}_{{\rm{pDIC}}}({\varTheta }_{{\rm{n}}},{Z}_{{\rm{n}}},t)$ is measured by pDIC on the outer surface and ${u}_{{\rm{in}}}({\varTheta }_{{\rm{n}}},{Z}_{{\rm{n}}},t)$ is computed using the OCT-DVC method so as to minimize, in a least-squares sense, the following quantity:

3 $$\mathop{\min }\limits_{{{\boldsymbol{u}}}_{{\rm{in}}}({\varTheta }_{{\rm{n}}},{Z}_{{\rm{n}}},t)}\mathop{\sum }\limits_{{\rm{q}}=1}^{{{\rm{N}}}_{{\rm{DVC}}}}{[{u}_{{\rm{DVC}}}({R}_{{\rm{q}}},{\varTheta }_{{\rm{q}}},{Z}_{{\rm{q}}},t)-\tilde{u}({R}_{{\rm{q}}},{\varTheta }_{{\rm{q}}},{Z}_{{\rm{q}}},t)]}^{2}$$

where the set of $({\varTheta }_{{\rm{n}}},{Z}_{{\rm{n}}})$ positions, with $\,1\le {\rm{n}}\le {{\rm{N}}}_{{\rm{pDIC}}},$ denote circumferential and axial coordinates of pDIC grid nodes on the outer surface. In contrast, $({R}_{{\rm{q}}},{\varTheta }_{{\rm{q}}},{Z}_{{\rm{q}}})$, with $1\le {\rm{q}}\le {{\rm{N}}}_{{\rm{DVC}}}$, denote the cylindrical coordinates of all measured OCT-DVC grid positions. Since uncertainty in the OCT-DVC results was estimated between 10 and 15 µm, ${{\rm{N}}}_{{\rm{pDIC}}}\ll {{\rm{N}}}_{{\rm{DVC}}}$ ensured a necessary and significant smoothing effect. Finally, the weight functions $\zeta (\varTheta -{\varTheta }_{{\rm{n}}},Z-{Z}_{{\rm{n}}})$ are derived from shape functions that ensure linear interpolation at a $(\varTheta ,Z)$ position from a set of $({\varTheta }_{{\rm{n}}},{Z}_{{\rm{n}}})$ positions. A similar process was performed for the in-plane displacement component $v$.

Since Eq. 3 only allows one to derive ${u}_{{\rm{in}}}({\varTheta }_{{\rm{n}}},{Z}_{{\rm{n}}},t)$ and ${v}_{{\rm{in}}}({\varTheta }_{{\rm{n}}},{Z}_{{\rm{n}}},t)$, we could not use OCT-DVC measurements to obtain $\tilde{w}({\boldsymbol{X}},t)$, which were not reliably available due to low image resolution. As the axial component of displacement usually has low variations in an inflation test, we assumed that it satisfied a Love-Kirchhoff beam equation at each cross section:

4 $$\tilde{w}({\boldsymbol{X}},t)={w}_{{\rm{av}}}(Z,t)+{{\rm{R}}}_{x}(Z,t)X+{{\rm{R}}}_{y}(Z,t)Y$$

with ${w}_{{\rm{av}}}(Z,t)$, ${{\rm{R}}}_{x}(Z,t)$ and ${{\rm{R}}}_{y}(Z,t)$ derived using a least-squares minimization against pDIC measurements. This provided a complete 3D reconstruction of displacement components $\tilde{u}$, $\tilde{v}$ and $\tilde{w}$ across $\Omega$. The referential gradients (for the $\tilde{u}$ and $\tilde{{\rm{v}}}$ components) were then directly obtained as:

5a $$\begin{array}{ccc}\frac{{\rm{\partial }}\mathop{u}\limits^{ \sim }}{{\rm{\partial }}X} & = & \mathop{\sum }\limits_{{\rm{n}}=1}^{{{\rm{N}}}_{{\rm{p}}{\rm{D}}{\rm{I}}{\rm{C}}}}\frac{-\frac{\frac{{\rm{\partial }}\zeta }{{\rm{\partial }}\varTheta }}{\sin \varTheta }\frac{(R-{R}_{{\rm{i}}{\rm{n}}}({\varTheta }_{{\rm{n}}},{Z}_{{\rm{n}}}))}{R}+\frac{\zeta }{\cos \varTheta }}{{R}_{{\rm{o}}{\rm{u}}{\rm{t}}}({\varTheta }_{{\rm{n}}},{Z}_{{\rm{n}}})-{R}_{{\rm{i}}{\rm{n}}}({\varTheta }_{{\rm{n}}},{Z}_{{\rm{n}}})}{u}_{{\rm{p}}{\rm{D}}{\rm{I}}{\rm{C}}}({\varTheta }_{{\rm{n}}},{Z}_{{\rm{n}}},t)\\  &  & -\mathop{\sum }\limits_{{\rm{n}}=1}^{{{\rm{N}}}_{{\rm{p}}{\rm{D}}{\rm{I}}{\rm{C}}}}\frac{\frac{\frac{{\rm{\partial }}\zeta }{{\rm{\partial }}\varTheta }}{\sin \varTheta }\frac{({R}_{{\rm{o}}{\rm{u}}{\rm{t}}}({\varTheta }_{{\rm{n}}},{Z}_{{\rm{n}}})-R)}{R}+\frac{\zeta }{\cos \varTheta }}{{R}_{{\rm{o}}{\rm{u}}{\rm{t}}}({\varTheta }_{{\rm{n}}},{Z}_{{\rm{n}}})-{R}_{{\rm{i}}{\rm{n}}}({\varTheta }_{{\rm{n}}},{Z}_{{\rm{n}}})}{u}_{{\rm{i}}{\rm{n}}}({\varTheta }_{{\rm{n}}},{Z}_{{\rm{n}}},t)\end{array}$$

5b $$\begin{array}{ccc}\frac{{\rm{\partial }}\mathop{u}\limits^{ \sim }}{{\rm{\partial }}Y} & = & \mathop{\sum }\limits_{n=1}^{{{\rm{N}}}_{{\rm{p}}{\rm{D}}{\rm{I}}{\rm{C}}}}\frac{\frac{\frac{{\rm{\partial }}\zeta }{{\rm{\partial }}\varTheta }}{\cos \varTheta }\frac{(R-{R}_{{\rm{i}}{\rm{n}}}({\varTheta }_{{\rm{n}}},{Z}_{{\rm{n}}}))}{R}+\frac{\zeta }{\sin \varTheta }}{{R}_{{\rm{o}}{\rm{u}}{\rm{t}}}({\varTheta }_{{\rm{n}}},{Z}_{{\rm{n}}})-{R}_{{\rm{i}}{\rm{n}}}({\varTheta }_{{\rm{n}}},{Z}_{{\rm{n}}})}{u}_{{\rm{p}}{\rm{D}}{\rm{I}}{\rm{C}}}({\varTheta }_{{\rm{n}}},{Z}_{{\rm{n}}},t)\\  &  & +\,\mathop{\sum }\limits_{{\rm{n}}=1}^{{{\rm{N}}}_{{\rm{p}}{\rm{D}}{\rm{I}}{\rm{C}}}}\frac{\frac{\frac{{\rm{\partial }}\zeta }{{\rm{\partial }}\varTheta }}{\cos \varTheta }\frac{({R}_{{\rm{o}}{\rm{u}}{\rm{t}}}({\varTheta }_{{\rm{n}}},{Z}_{{\rm{n}}})-R)}{R}-\frac{\zeta }{\sin \varTheta }}{{R}_{{\rm{o}}{\rm{u}}{\rm{t}}}({\varTheta }_{{\rm{n}}},{Z}_{{\rm{n}}})-{R}_{{\rm{i}}{\rm{n}}}({\varTheta }_{{\rm{n}}},{Z}_{{\rm{n}}})}{u}_{{\rm{i}}{\rm{n}}}({\varTheta }_{{\rm{n}}},{Z}_{{\rm{n}}},t)\end{array}$$

5c $$\begin{array}{ccc}\frac{{\rm{\partial }}\mathop{u}\limits^{ \sim }}{{\rm{\partial }}Z} & = & \mathop{\sum }\limits_{n=1}^{{{\rm{N}}}_{{\rm{p}}{\rm{D}}{\rm{I}}{\rm{C}}}}\frac{{\rm{\partial }}\zeta }{{\rm{\partial }}Z}\frac{(R-{R}_{{\rm{i}}{\rm{n}}}({\varTheta }_{{\rm{n}}},{Z}_{{\rm{n}}}))}{{R}_{{\rm{o}}{\rm{u}}{\rm{t}}}({\varTheta }_{{\rm{n}}},{Z}_{{\rm{n}}})-{R}_{{\rm{i}}{\rm{n}}}({\varTheta }_{{\rm{n}}},{Z}_{{\rm{n}}})}{u}_{{\rm{p}}{\rm{D}}{\rm{I}}{\rm{C}}}({\varTheta }_{{\rm{n}}},{Z}_{{\rm{n}}},t)\\  &  & +\,\mathop{\sum }\limits_{{\rm{n}}=1}^{{{\rm{N}}}_{{\rm{p}}{\rm{D}}{\rm{I}}{\rm{C}}}}\frac{{\rm{\partial }}\zeta }{{\rm{\partial }}Z}\frac{({R}_{{\rm{o}}{\rm{u}}{\rm{t}}}({\varTheta }_{{\rm{n}}},{Z}_{{\rm{n}}})-R)}{{R}_{{\rm{o}}{\rm{u}}{\rm{t}}}({\varTheta }_{{\rm{n}}},{Z}_{{\rm{n}}})-{R}_{{\rm{i}}{\rm{n}}}({\varTheta }_{{\rm{n}}},{Z}_{{\rm{n}}})}{u}_{{\rm{i}}{\rm{n}}}({\varTheta }_{{\rm{n}}},{Z}_{{\rm{n}}},t).\end{array}$$

The gradients for the $\tilde{w}$ component were obtained directly from $Z$ differentiation of Eq. 4. Eventually, the deformation gradient was derived from the displacement gradients at every position across ${\Omega }$ as:

6 $$\tilde{{\boldsymbol{F}}}({\boldsymbol{X}},t)=\left[\begin{array}{ccc}1+\frac{\partial \tilde{u}}{\partial X} & \frac{\partial \tilde{{\rm{u}}}}{\partial Y} & \frac{\partial \tilde{u}}{\partial Z}\\ \frac{\partial \tilde{v}}{\partial X} & 1+\frac{\partial \tilde{v}}{\partial Y} & \frac{\partial \tilde{v}}{\partial Z}\\ \frac{\partial \tilde{w}}{\partial X} & \frac{\partial \tilde{w}}{\partial Y} & 1+\frac{\partial \tilde{w}}{\partial Z}\end{array}\right]$$

Thus, using data from pDIC and OCT-DVC – and making assumptions about the spatial distributions of the pointwise displacement fields – we were able to reconstruct a continuous deformation gradient field across $\Omega$, for any configuration $t$, denoted $\tilde{{\boldsymbol{F}}}({\boldsymbol{X}},t)$.

### Material model

Prior experience revealed that a microstructurally motivated constrained mixture model describes the constitutive behavior of murine arteries well^57^. For every material position ***X***, such a hyperelastic model was considered, namely, a strain energy function, defined per unit volume, of the form:

7 $$W({\boldsymbol{X}})=U(J)+{{\mathbb{I}}}_{{\Omega }_{{\rm{w}}{\rm{a}}{\rm{l}}{\rm{l}}}}({\boldsymbol{X}})\mathop{\sum }\limits_{i=1}^{4}{W}^{{\rm{i}}}(\bar{{\boldsymbol{F}}})+{{\mathbb{I}}}_{{\Omega }_{{\rm{i}}{\rm{m}}{\rm{t}}}}({\boldsymbol{X}}){W}^{5}(\bar{{\boldsymbol{F}}})$$

where ${{\mathbb{I}}}_{{{\Omega }}_{{\rm{wall}}}}({\boldsymbol{X}})=1$ when ${\boldsymbol{X}}\in {{\Omega }}_{{\rm{wall}}}$ and ${{\mathbb{I}}}_{{\Omega }_{{\rm{w}}{\rm{a}}{\rm{l}}{\rm{l}}}}({\boldsymbol{X}})=0$ otherwise. ${{\mathbb{I}}}_{{{\Omega }}_{{\rm{imt}}}}$ is defined similarly for the intramural thrombus, $J={\rm{\det }}(\tilde{{\boldsymbol{F}}})$ and $\bar{{\boldsymbol{F}}}=\tilde{{\boldsymbol{F}}}/{J}^{1/3}$, with $U(J)$ the strain energy related to volume change. The ${W}^{{\rm{i}}}$ quantities are strain energy densities per unit volume defined as

8a $${W}^{1}(\bar{{\boldsymbol{F}}})=\frac{{{\rm{c}}}^{1}}{4{{\rm{k}}}^{1}}[[{e}^{{{\rm{k}}}^{1}{|({(\bar{{\boldsymbol{F}}}{{\boldsymbol{G}}}_{1})}^{T}(\bar{{\boldsymbol{F}}}{{\boldsymbol{G}}}_{1}^{})):{{\bf{A}}}_{1}-1|}_{+}^{2}}-1]+\alpha [{e}^{{{\rm{k}}}^{1}{|({(\bar{{\boldsymbol{F}}}{{\boldsymbol{G}}}_{1})}^{T}(\bar{{\boldsymbol{F}}}{{\boldsymbol{G}}}_{1})):{{\bf{A}}}_{1}-1|}_{-}^{2}}-1]]$$

8b $${W}^{2}(\bar{{\boldsymbol{F}}})=\frac{{{\rm{c}}}^{2}}{4{{\rm{k}}}^{2}}[[{e}^{{{\rm{k}}}^{2}{|({(\bar{{\boldsymbol{F}}}{{\boldsymbol{G}}}_{2})}^{T}(\bar{{\boldsymbol{F}}}{{\boldsymbol{G}}}_{2})):{{\bf{A}}}_{2}-1|}_{+}^{2}}-1]+\alpha [{e}^{{{\rm{k}}}^{2}{|({(\bar{{\boldsymbol{F}}}{{\boldsymbol{G}}}_{2})}^{T}(\bar{{\boldsymbol{F}}}{{\boldsymbol{G}}}_{2}^{})):{{\bf{A}}}_{2}-1|}_{-}^{2}}-1]]$$

8c $$\begin{array}{rcl}{W}^{3}(\bar{{\boldsymbol{F}}}) & = & \frac{{{\rm{c}}}^{3}}{4{{\rm{k}}}^{3}}\{[[{e}^{{{\rm{k}}}^{3}{|({(\bar{{\boldsymbol{F}}}{{\boldsymbol{G}}}_{3}^{+})}^{T}(\bar{{\boldsymbol{F}}}{{\boldsymbol{G}}}_{3}^{+})):{{\bf{A}}}_{3}^{+}-1|}_{+}^{2}}-1]+\alpha [{e}^{{{\rm{k}}}^{3}{|({(\bar{{\boldsymbol{F}}}{{\boldsymbol{G}}}_{3}^{+})}^{T}(\bar{{\boldsymbol{F}}}{{\boldsymbol{G}}}_{3}^{+3})):{{\bf{A}}}_{3}^{+}-1|}_{-}^{2}}-1]]\\  &  & +\,[[{e}^{{{\rm{k}}}^{3}{|({(\bar{{\boldsymbol{F}}}{{\boldsymbol{G}}}_{3}^{-})}^{T}(\bar{{\boldsymbol{F}}}{{\boldsymbol{G}}}_{3}^{-})):{{\bf{A}}}_{3}^{-}-1|}_{+}^{2}}-1]+\alpha [{e}^{{{\rm{k}}}^{3}{|({(\bar{{\boldsymbol{F}}}{{\boldsymbol{G}}}_{3}^{-})}^{T}(\bar{{\boldsymbol{F}}}{{\boldsymbol{G}}}_{3}^{-})):{{\bf{A}}}_{3}^{-}-1|}_{-}^{2}}-1]]\}\end{array}$$

8d $${W}^{4}(\bar{{\boldsymbol{F}}})=\frac{{{\rm{c}}}^{4}}{2}[({(\bar{{\boldsymbol{F}}}{{\boldsymbol{G}}}_{4})}^{T}(\bar{{\boldsymbol{F}}}{{\boldsymbol{G}}}_{4})):{\bf{I}}-3]$$

8e $${W}^{5}(\bar{{\boldsymbol{F}}})=\frac{{{\rm{c}}}^{5}}{2}[{\rm{tr}}({\bar{{\boldsymbol{F}}}}^{T}\bar{{\boldsymbol{F}}})-3].$$

Note that *W*^4^ is a neoHookean strain energy density function for the elastin-dominated matrix, *W*^5^ is a neoHookean strain energy function for the thrombus, and ${W}^{{\rm{i}}}(1\le {\rm{i}}\le 3)$ are coordinate invariant-based Fung-type exponential strain energy functions which model the circumferential, axial, and diagonal contributions of the fibrous tissue network, respectively^5^. Quantities ${{\rm{c}}}^{{\rm{i}}}(1\le {\rm{i}}\le 5)$ and ${{\rm{k}}}^{{\rm{i}}}\,(1\le {\rm{i}}\le 3)$ are material parameters and ${\rm{\alpha }}$ $(0\le {\rm{\alpha }}\le 1)$ is a ratio that accounts for the differential contribution of fibers in compression and tension.

The ${{\bf{A}}}_{{\rm{i}}}^{+/-}$ are tensors defined as: ${{\bf{A}}}_{{\rm{i}}}^{+/-}={{\bf{a}}}_{{\rm{i}}}^{+/-}\otimes {{\bf{a}}}_{{\rm{i}}}^{+/-}$, where ${{\bf{a}}}_{{\rm{i}}}^{+/-}$ indicates the vector for the direction of a given fiber family (${{\bf{a}}}_{1}^{+}={{\bf{a}}}_{1}^{-}={{\bf{a}}}_{1}$ and ${{\bf{a}}}_{2}^{+}={{\bf{a}}}_{2}^{-}={{\bf{a}}}_{2}$). The four fiber families considered in the current model are given by the following directions:

9a $${{\bf{a}}}_{1}={{\bf{e}}}_{{\boldsymbol{c}}}$$

9b $${{\bf{a}}}_{2}={{\bf{e}}}_{{\boldsymbol{a}}}$$

9c $${{\bf{a}}}_{3}^{+}=\,\cos \,{\rm{\beta }}{{\bf{e}}}_{{\rm{c}}}+\,\sin \,{\rm{\beta }}{{\bf{e}}}_{{\rm{a}}}$$

9d $${{\bf{a}}}_{3}^{-}=\,\cos \,{\rm{\beta }}{{\bf{e}}}_{{\rm{c}}}-\,\sin \,{\rm{\beta }}{{\bf{e}}}_{{\rm{a}}}$$

where ${\rm{\beta }}$ is a material parameter. Vectors ${{\bf{e}}}_{{\rm{c}}}$, ${{\bf{e}}}_{{\rm{a}}}$ and ${{\bf{e}}}_{{\rm{o}}}$ define the material local basis, where ${{\bf{e}}}_{{\rm{a}}}$ is aligned with the centerline of the artery, ${{\bf{e}}}_{{\rm{o}}}$ is obtained by solving the Laplace equation across $\Omega$ ($\Delta \xi =0$), with Dirichlet boundary conditions ($\xi =0$ across ${S}_{{\rm{i}}{\rm{n}}}$ and $\xi =1$ across ${S}_{{\rm{o}}{\rm{u}}{\rm{t}}}$). Then at every position, ${{\bf{e}}}_{{\rm{o}}}$ is the same direction as $\nabla \xi$. Finally, ${{\bf{e}}}_{{\rm{c}}}$ = ${{\bf{e}}}_{{\rm{a}}}$ × ${{\bf{e}}}_{{\rm{o}}}$.

The ${{\boldsymbol{G}}}_{{\rm{i}}}^{+/-}$ $(1\le {\rm{i}}\le 4)$ terms are deposition stretch tensors, defined as

10a $${{\boldsymbol{G}}}_{1}={{\rm{G}}}^{1}{{\bf{a}}}_{1}\otimes {{\bf{a}}}_{1}+\frac{1}{{{\rm{G}}}^{1}}({\bf{I}}-{{\bf{a}}}_{1}\otimes {{\bf{a}}}_{1})$$

10b $${{\bf{G}}}_{2}={{\rm{G}}}^{2}{{\bf{a}}}_{2}\otimes {{\bf{a}}}_{2}+\frac{1}{{{\rm{G}}}^{2}}({\bf{I}}-{{\bf{a}}}_{2}\otimes {{\bf{a}}}_{2})$$

10c $${{\bf{G}}}_{3}^{+}={{\rm{G}}}^{3}{{\bf{a}}}_{3}^{+}\otimes {{\bf{a}}}_{3}^{+}+\frac{1}{{{\rm{G}}}^{3}}({\bf{I}}-{{\bf{a}}}_{3}^{+}\otimes {{\bf{a}}}_{3}^{+})$$

10d $${{\bf{G}}}_{3}^{-}={{\rm{G}}}^{3}{{\bf{a}}}_{3}^{-}\otimes {{\bf{a}}}_{3}^{-}+\frac{1}{{{\rm{G}}}^{3}}({\bf{I}}-{{\bf{a}}}_{3}^{-}\otimes {{\bf{a}}}_{3}^{-})$$

10e $${{\bf{G}}}_{4}={{\rm{G}}}^{4}{{\bf{e}}}_{{\boldsymbol{c}}}\otimes {{\bf{e}}}_{{\boldsymbol{c}}}+{{\rm{G}}}^{a}{{\bf{e}}}_{{\boldsymbol{a}}}\otimes {{\bf{e}}}_{{\boldsymbol{a}}}+\frac{1}{{{\rm{G}}}^{4}{{\rm{G}}}^{a}}{{\bf{e}}}_{{\boldsymbol{o}}}\otimes {{\bf{e}}}_{{\boldsymbol{o}}}$$

where ${{\rm{G}}}^{{\rm{i}}}(1\le {\rm{i}}\le 4)$ are deposition stretch values (i.e., stretches at which constituents are incorporated within extant matrix) and, with the appropriate material properties, satisfy equilibrium in the reference state. ${{\rm{G}}}^{a}$ is the average axial stretch of the artery with respect to its stress-free configuration.

We assume that ${\rm{\alpha }}$, ${\rm{\beta }}$, ${{\rm{c}}}^{{\rm{i}}}(1\le {\rm{i}}\le 5)$, ${{\rm{G}}}^{{\rm{i}}}(1\le {\rm{i}}\le 4)$ and ${{\rm{k}}}^{{\rm{i}}}(1\le {\rm{i}}\le 3)$ all vary spatially, meaning they are functions of ${\boldsymbol{X}}$: ${\rm{\alpha }}({\boldsymbol{X}}),\,{\rm{\beta }}({\boldsymbol{X}})$, ${{\rm{c}}}^{{\rm{i}}}({\boldsymbol{X}}),{{\rm{G}}}^{{\rm{i}}}({\boldsymbol{X}}),{{\rm{k}}}^{{\rm{i}}}({\boldsymbol{X}})$. Following previous experience, we assume that ${G}^{a}$ is constant across the domain. Additionally, we neglect radial variations of material properties in Ω_wall_. We define a partition of the domain of interest $\Omega \,{\rm{i}}{\rm{n}}\,{{\rm{N}}}_{\varTheta }{{\rm{N}}}_{Z}$ hexahedra, with ${{\rm{N}}}_{\varTheta }$ angular sectors and ${{\rm{N}}}_{Z}$ axial segments. Each hexahedron has a rectangular face on the inner surface and another one on the outer surface; it is denoted with its angular and axial positions $({{\rm{i}}}_{\varTheta },{{\rm{i}}}_{Z})$. For any of the unknown material properties, we assume:

11a $${{\rm{c}}}^{{\rm{i}}}({\boldsymbol{X}})=\mathop{\sum }\limits_{{{\rm{i}}}_{Z}=0}^{{{\rm{N}}}_{Z}}\mathop{\sum }\limits_{{{\rm{i}}}_{\varTheta }=0}^{{{\rm{N}}}_{\varTheta }}{{\rm{c}}}_{{{\rm{i}}}_{\varTheta },{{\rm{i}}}_{Z}}^{{\rm{i}}}{{\mathbb{I}}}_{{{\rm{i}}}_{\varTheta },{{\rm{i}}}_{Z}}({\boldsymbol{X}})$$

11b $${\Xi }^{{\rm{i}}}({\boldsymbol{X}})=\mathop{\sum }\limits_{{{\rm{k}}}_{Z}=0}^{{{\rm{N}}}_{Z}}\mathop{\sum }\limits_{{{\rm{i}}}_{\varTheta }=0}^{{{\rm{N}}}_{\varTheta }}{\Xi }_{{{\rm{i}}}_{\varTheta },{{\rm{i}}}_{Z}}^{{\rm{i}}}{{\mathbb{I}}}_{{{\rm{i}}}_{\varTheta },{{\rm{i}}}_{Z}}({\boldsymbol{X}})$$

where ${\Xi }^{{\rm{i}}}={{\rm{k}}}^{{\rm{i}}}$ for $1\le {\rm{i}}\le 3$, ${\Xi }^{{\rm{i}}}={{\rm{G}}}^{{\rm{i}}-3}$ for $4\le {\rm{i}}\le 7$, ${\Xi }^{{\rm{i}}}={\rm{\beta }}$ for ${\rm{i}}=8$, and ${\Xi }^{{\rm{i}}}={\rm{\alpha }}$ for ${\rm{i}}=9$. The ${{\rm{c}}}_{{{\rm{i}}}_{\varTheta },{{\rm{i}}}_{Z}}^{{\rm{i}}}$ parameters are those upon which the strain energy density depends linearly, whereas the ${\Xi }_{{{\rm{i}}}_{\varTheta },{{\rm{i}}}_{Z}}^{{\rm{i}}}$ parameters are those upon which the strain energy density depends nonlinearly. Note that ${{\mathbb{I}}}_{{{\rm{i}}}_{\varTheta },{{\rm{i}}}_{Z}}({\boldsymbol{X}})$ = 1 when ${\boldsymbol{x}}$ belongs to the $({{\rm{i}}}_{\varTheta },{{\rm{i}}}_{Z})$ hexahedron (further denoted ${\Omega }_{\,}^{{{\rm{i}}}_{\varTheta },{{\rm{i}}}_{Z}}$), and ${{\mathbb{I}}}_{{{\rm{i}}}_{\varTheta },{{\rm{i}}}_{Z}}({\boldsymbol{X}})=0$ otherwise. This leads to the identification of ~14 ${{\rm{N}}}_{\varTheta }{{\rm{N}}}_{Z}$ material properties (only 13 unknowns in hexahedra that do not include thrombus), consisting of 5 ${N}_{\varTheta }{N}_{Z}$ linear parameters ${{\rm{c}}}_{{{\rm{i}}}_{\varTheta },{{\rm{i}}}_{Z}}^{{\rm{i}}}$ and 9 ${N}_{\varTheta }{N}_{Z}$ nonlinear parameters ${\Xi }_{{{\rm{i}}}_{\varTheta },{{\rm{i}}}_{Z}}^{{\rm{i}}}$.

Following inverse characterization of these ~14 ${N}_{\varTheta }{N}_{Z}$ unknowns, we can easily compute the full-field distributions of key mechanical metrics such as stored energy, circumferential material stiffness (linearized about the pressure of interest), and circumferential stress. In particular, stored energy and material stiffness represent collective effects of the identified set of material parameters; values reported herein were computed at the specimen-specific value of the *in vivo* axial stretch and a distending pressure of either 140 mmHg (near *in vivo* pressure values for this hypertensive mouse model) or 80 mmHg (the reference state).

### Inverse Material Characterization

The Cauchy stress can be written at position ${\boldsymbol{x}}$ = ${\boldsymbol{X}}+{\boldsymbol{u}}$ such that:

12 $$\begin{array}{ccc}{\boldsymbol{\sigma }}({\boldsymbol{x}}) & = & -\frac{{\rm{\partial }}U}{{\rm{\partial }}J}{\boldsymbol{I}}+\mathop{\sum }\limits_{{{\rm{i}}}_{Z}=0}^{{{\rm{N}}}_{Z}}\mathop{\sum }\limits_{{{\rm{i}}}_{\varTheta }=0}^{{{\rm{N}}}_{\varTheta }}{J}^{-1}[{{\mathbb{I}}}_{{{\rm{i}}}_{\varTheta },{{\rm{i}}}_{Z}}^{{\rm{w}}{\rm{a}}{\rm{l}}{\rm{l}}}({\boldsymbol{x}})\mathop{\sum }\limits_{i=1}^{3}{{\rm{c}}}_{{{\rm{i}}}_{\varTheta },{{\rm{i}}}_{Z}}^{{\rm{i}}}{\psi }^{{\rm{i}}}{\rm{d}}{\rm{e}}{\rm{v}}((\bar{{\boldsymbol{F}}}{{\boldsymbol{G}}}_{{\rm{i}}}{{\bf{a}}}_{{\rm{i}}})\,(\bar{{\boldsymbol{F}}}{{\boldsymbol{G}}}_{{\rm{i}}}{{\bf{a}}}_{{\rm{i}}}))\\  &  & +{{\mathbb{I}}}_{{{\rm{i}}}_{\varTheta },{{\rm{i}}}_{Z}}^{{\rm{w}}{\rm{a}}{\rm{l}}{\rm{l}}}({\boldsymbol{x}}){{\rm{c}}}_{{{\rm{i}}}_{\varTheta },{{\rm{i}}}_{Z}}^{4}{\rm{d}}{\rm{e}}{\rm{v}}((\bar{{\boldsymbol{F}}}{{\boldsymbol{G}}}_{4}){(\bar{{\boldsymbol{F}}}{{\boldsymbol{G}}}_{4})}^{T})+{{\mathbb{I}}}_{{{\rm{i}}}_{\varTheta },{{\rm{i}}}_{Z}}^{{\rm{i}}{\rm{m}}{\rm{t}}}({\boldsymbol{x}}){{\rm{c}}}_{{{\rm{i}}}_{\varTheta },{{\rm{i}}}_{Z}}^{5}{\rm{d}}{\rm{e}}{\rm{v}}(\bar{{\boldsymbol{F}}}{\bar{{\boldsymbol{F}}}}^{T})]\end{array}$$

where ${{\bf{a}}}_{3}$ may be either ${{\bf{a}}}_{3}^{+}$ or ${{\bf{a}}}_{3}^{-}$ values, and

13 $$\begin{array}{c}{\psi }^{{\rm{i}}}={|({(\bar{{\boldsymbol{F}}}{{\boldsymbol{G}}}_{{\rm{i}}})}^{T}(\bar{{\boldsymbol{F}}}{{\boldsymbol{G}}}_{{\rm{i}}})):{{\boldsymbol{A}}}_{{\rm{i}}}-1|}_{+}{e}^{{{\rm{k}}}^{{\rm{i}}}({\boldsymbol{x}}){|({(\bar{{\boldsymbol{F}}}{{\boldsymbol{G}}}_{{\rm{i}}})}^{T}(\bar{{\boldsymbol{F}}}{{\boldsymbol{G}}}_{{\rm{i}}})):{{\boldsymbol{A}}}_{{\bf{i}}}-1|}_{+}^{2}}\\ \,+\alpha ({\boldsymbol{x}}){|({(\bar{{\boldsymbol{F}}}{{\boldsymbol{G}}}_{{\rm{i}}})}^{T}(\bar{{\boldsymbol{F}}}{{\boldsymbol{G}}}_{{\rm{i}}})):{{\bf{A}}}_{{\rm{i}}}-1|}_{-}{e}^{{{\rm{k}}}^{{\rm{i}}}({\boldsymbol{x}}){|({(\bar{{\boldsymbol{F}}}{{\boldsymbol{G}}}_{{\rm{i}}})}^{T}(\bar{{\boldsymbol{F}}}{{\boldsymbol{G}}}_{{\rm{i}}})):{{\boldsymbol{A}}}_{{\bf{i}}}-1|}_{-}^{2}}\end{array}$$

The principle of virtual power (PVP) was used to identify the ~14 ${{\rm{N}}}_{\varTheta }{{\rm{N}}}_{Z}$ unknown material parameters^72^. The PVP is an integral expression of the equilibrium equations for a solid, which may be written

14 $$\mathop{\underbrace{-\,{\int }_{\Omega (t)}{\boldsymbol{\sigma }}:{{\boldsymbol{\varepsilon }}}^{\ast }\,d{\boldsymbol{x}}}}\limits_{-{P}_{{\rm{i}}{\rm{n}}{\rm{t}}}^{\ast }(t)}+\mathop{\underbrace{{\oint }_{{\rm{\partial }}\Omega (t)}{\boldsymbol{T}}.{{\boldsymbol{u}}}^{\ast }\,d{\boldsymbol{x}}}}\limits_{{P}_{{\rm{e}}{\rm{x}}{\rm{t}}}^{\ast }(t)}=0$$

where ${{\boldsymbol{u}}}^{\ast }$ is a virtual velocity field defined over volume in a deformed configuration and ${{\boldsymbol{\varepsilon }}}^{\ast }$ is the virtual rate of deformation deduced from velocity ${{\boldsymbol{u}}}^{\ast }$ according to:

15 $${{\boldsymbol{\varepsilon }}}^{\ast }=\frac{1}{2}(\nabla {{\boldsymbol{u}}}^{\ast }+{\nabla }^{{\boldsymbol{T}}}{{\boldsymbol{u}}}^{\ast })$$

where ***T*** are the tractions across the boundary, ${P}_{{\rm{int}}}^{\ast }$ is the virtual power of internal forces, and ${P}_{{\rm{ext}}}^{\ast }$ is the virtual power of external forces.

In the following, we use only virtual fields such that ${\boldsymbol{I}}:{{\boldsymbol{\varepsilon }}}^{\ast }=0$. By combining Eq. 12 and Eq. 14, the virtual power of internal forces may be written:

16 $$\begin{array}{ccc}{P}_{{\rm{i}}{\rm{n}}{\rm{t}}}^{\ast }(t) & = & \mathop{\sum }\limits_{{{\rm{i}}}_{Z}=0}^{{{\rm{N}}}_{Z}}\mathop{\sum }\limits_{{{\rm{i}}}_{\varTheta }=0}^{{{\rm{N}}}_{\varTheta }}[\mathop{\sum }\limits_{i=1}^{3}{{\rm{c}}}_{{{\rm{i}}}_{\varTheta },{{\rm{i}}}_{Z}}^{{\rm{i}}}{\int }_{{\Omega }_{{\rm{w}}{\rm{a}}{\rm{l}}{\rm{l}}}^{{{\rm{i}}}_{\varTheta },{{\rm{i}}}_{Z}}}{J}^{-1}{\psi }^{{\rm{i}}}{\rm{d}}{\rm{e}}{\rm{v}}(({\boldsymbol{F}}{{\boldsymbol{G}}}_{{\rm{i}}}{{\bf{a}}}_{{\rm{i}}})\otimes ({\boldsymbol{F}}{{\boldsymbol{G}}}_{{\rm{i}}}{{\bf{a}}}_{{\rm{i}}})):\,{{\boldsymbol{\varepsilon }}}^{\ast }d{\boldsymbol{x}}\\  &  & +{{\rm{c}}}_{{{\rm{i}}}_{\varTheta },{{\rm{i}}}_{Z}}^{4}{\int }_{{\Omega }_{{\rm{w}}{\rm{a}}{\rm{l}}{\rm{l}}}^{{{\rm{i}}}_{\varTheta },{{\rm{i}}}_{Z}}}{J}^{-1}\,{\rm{d}}{\rm{e}}{\rm{v}}(({\boldsymbol{F}}{{\boldsymbol{G}}}_{4}){({\boldsymbol{F}}{{\boldsymbol{G}}}_{4})}^{T}):\,{{\boldsymbol{\varepsilon }}}^{\ast }d{\boldsymbol{x}}+{{\rm{c}}}_{{{\rm{i}}}_{\varTheta },{{\rm{i}}}_{Z}}^{5}{\int }_{{\Omega }_{{\rm{i}}{\rm{m}}{\rm{t}}}^{{{\rm{i}}}_{\varTheta },{{\rm{i}}}_{Z}}}{J}^{-1}\,{\rm{d}}{\rm{e}}{\rm{v}}({\boldsymbol{F}}{{\boldsymbol{F}}}^{T}):{{\boldsymbol{\varepsilon }}}^{\ast }d{\boldsymbol{x}}].\end{array}$$

We define exactly $2{{\rm{N}}}_{\varTheta }{{\rm{N}}}_{Z}$ different virtual fields for $1\le {{\rm{j}}}_{\varTheta }\le {\rm{N}}$ and $1\le {{\rm{j}}}_{Z}\le {{\rm{N}}}_{Z}$, such that:

17a $${{\boldsymbol{u}}}_{{{\rm{j}}}_{\varTheta },{{\rm{j}}}_{Z}}^{\ast (1)}({\boldsymbol{x}})=\frac{1}{{p}_{{\rm{ref}}}}\left[\frac{x}{{x}^{2}+{y}^{2}}\,{\boldsymbol{i}}+\frac{y}{{x}^{2}+{y}^{2}}\,{\boldsymbol{j}}\right]W(\theta -{\theta }_{{{\rm{j}}}_{\varTheta }},z-{z}_{{{\rm{j}}}_{Z}})$$

17b $${{\boldsymbol{u}}}_{{{\rm{j}}}_{\varTheta },{{\rm{j}}}_{Z}}^{\ast (2)}({\boldsymbol{x}})=\frac{1}{{F}_{{\rm{ref}}}}\left(-\frac{x}{2}{\boldsymbol{i}}-\frac{y}{2}\,{\boldsymbol{j}}+z\,{\boldsymbol{k}}\right)W(\theta -{\theta }_{{{\rm{j}}}_{\varTheta }},z-{z}_{{{\rm{j}}}_{Z}}),$$

where $({\theta }_{{{\rm{j}}}_{\varTheta }},{z}_{{{\rm{j}}}_{Z}})$ denotes the deformed angular and axial position of the ${\Omega }^{{{\rm{j}}}_{\varTheta },{{\rm{j}}}_{Z}}$ hexahedron $({\varTheta }_{{{\rm{j}}}_{\varTheta }},{Z}_{{{\rm{j}}}_{Z}})$ at time *t*, and ${p}_{{\rm{ref}}}$ and ${F}_{{\rm{ref}}}$ are the intraluminal pressure and axial force at the *in vivo* reference configuration, respectively; they are used for normalization, ensuring virtual powers of the same order of magnitude for both virtual fields. The *W* function is defined as:

18 $$W(\theta -{\theta }_{{{\rm{j}}}_{\varTheta }},z-{z}_{{{\rm{j}}}_{Z}})=\exp \left(-,\frac{{(\theta -{\theta }_{{{\rm{j}}}_{\varTheta }})}^{2}}{10\pi /{{\rm{N}}}_{\varTheta }},-,\frac{{(z-{z}_{{{\rm{j}}}_{Z}})}^{2}}{5({Z}_{{\rm{u}}{\rm{p}}}-{Z}_{{\rm{d}}{\rm{o}}{\rm{w}}{\rm{n}}}\,)/{{\rm{N}}}_{Z}}\right)$$

and the virtual strain fields are

19a $${\varepsilon }_{xx-{{\rm{j}}}_{\varTheta },{{\rm{j}}}_{Z}}^{\ast (1)}({\boldsymbol{x}})=\frac{{y}^{2}-{x}^{2}}{{({x}^{2}+{y}^{2})}^{2}}W(\theta -{\theta }_{{{\rm{j}}}_{\varTheta }},z-{z}_{{{\rm{j}}}_{Z}})-\frac{xy}{{({x}^{2}+{y}^{2})}^{2}}\,\frac{\partial W}{\partial \theta }(\theta -{\theta }_{{{\rm{j}}}_{\varTheta }},z-{z}_{{{\rm{j}}}_{Z}})$$

19b $${\varepsilon }_{yy-{{\rm{j}}}_{\varTheta },{{\rm{j}}}_{Z}}^{\ast (1)}({\boldsymbol{x}})=\frac{{x}^{2}-{y}^{2}}{{({x}^{2}+{y}^{2})}^{2}}W(\theta -{\theta }_{{{\rm{j}}}_{\varTheta }},z-{z}_{{{\rm{j}}}_{Z}})+\frac{xy}{{({x}^{2}+{y}^{2})}^{2}}\,\frac{\partial W}{\partial \theta }(\theta -{\theta }_{{{\rm{j}}}_{\varTheta }},z-{z}_{{{\rm{j}}}_{Z}})$$

19c $${\varepsilon }_{zz-{{\rm{j}}}_{\varTheta },{{\rm{j}}}_{Z}}^{\ast (1)}({\boldsymbol{x}})=0$$

19d $${\varepsilon }_{xy-{{\rm{j}}}_{\varTheta },{{\rm{j}}}_{Z}}^{\ast (1)}({\boldsymbol{x}})=\frac{2xy}{{({x}^{2}+{y}^{2})}^{2}}W(\theta -{\theta }_{{{\rm{j}}}_{\varTheta }},z-{z}_{{{\rm{j}}}_{Z}})+\frac{({x}^{2}-{y}^{2})/2}{{({x}^{2}+{y}^{2})}^{2}}\,\frac{{\rm{\partial }}W}{{\rm{\partial }}\theta }(\theta -{\theta }_{{{\rm{j}}}_{\varTheta }},z-{z}_{{{\rm{j}}}_{Z}})$$

19e $${\varepsilon }_{xz-{{\rm{j}}}_{\varTheta },{{\rm{j}}}_{Z}}^{\ast (1)}({\boldsymbol{x}})=\frac{1}{2}\frac{x}{{x}^{2}+{y}^{2}}\,\frac{\partial W}{\partial z}(\theta -{\theta }_{{{\rm{j}}}_{\varTheta }},z-{z}_{{{\rm{j}}}_{Z}})$$

19f $${\varepsilon }_{yz-{{\rm{j}}}_{\varTheta },{{\rm{j}}}_{Z}}^{\ast (1)}({\boldsymbol{x}})=\frac{1}{2}\frac{y}{{x}^{2}+{y}^{2}}\,\frac{\partial W}{\partial z}(\theta -{\theta }_{{{\rm{j}}}_{\varTheta }},z-{z}_{{{\rm{j}}}_{Z}})$$

20a $${\varepsilon }_{xx-{{\rm{j}}}_{\varTheta },{{\rm{j}}}_{Z}}^{\ast (2)}({\boldsymbol{x}})=-\frac{1}{2}W(\theta -{\theta }_{{{\rm{j}}}_{\varTheta }},z-{z}_{{{\rm{j}}}_{Z}})-\frac{xy}{2({x}^{2}+{y}^{2})}\,\frac{\partial W}{\partial \theta }(\theta -{\theta }_{{{\rm{j}}}_{\varTheta }},z-{z}_{{{\rm{j}}}_{Z}})$$

20b $${\varepsilon }_{yy-{{\rm{j}}}_{\varTheta },{{\rm{j}}}_{Z}}^{\ast (2)}({\boldsymbol{x}})=-\frac{1}{2}W(\theta -{\theta }_{{{\rm{j}}}_{\varTheta }},z-{z}_{{{\rm{j}}}_{Z}})-\frac{xy}{2({x}^{2}+{y}^{2})}\,\frac{\partial W}{\partial \theta }(\theta -{\theta }_{{{\rm{j}}}_{\varTheta }},z-{z}_{{{\rm{j}}}_{Z}})$$

20c $${\varepsilon }_{zz-{{\rm{j}}}_{\varTheta },{{\rm{j}}}_{Z}}^{\ast (2)}({\boldsymbol{x}})=W(\theta -{\theta }_{{{\rm{j}}}_{\varTheta }},z-{z}_{{{\rm{j}}}_{Z}})+\frac{xy}{({x}^{2}+{y}^{2})}\,\frac{\partial W}{\partial \theta }(\theta -{\theta }_{{{\rm{j}}}_{\varTheta }},z-{z}_{{{\rm{j}}}_{Z}})$$

20d $${\varepsilon }_{xy-{\rm{j}},{{\rm{j}}}_{Z}}^{\ast (2)}({\boldsymbol{x}})=-\frac{1}{4}\,\frac{\partial W}{\partial \theta }(\theta -{\theta }_{{\rm{j}}},z-{z}_{{{\rm{j}}}_{Z}})$$

20e $${\varepsilon }_{xz-{{\rm{j}}}_{\varTheta },{{\rm{j}}}_{Z}}^{\ast (2)}({\boldsymbol{x}})=-\frac{yz}{2({x}^{2}+{y}^{2})}\frac{\partial W}{\partial \theta }(\theta -{\theta }_{{{\rm{j}}}_{\varTheta }},z-{z}_{{{\rm{j}}}_{Z}})+\frac{z}{2}\frac{\partial W}{\partial z}(\theta -{\theta }_{{{\rm{j}}}_{\varTheta }},z-{z}_{{{\rm{j}}}_{Z}})$$

20f $${\varepsilon }_{yz-{{\rm{j}}}_{\varTheta },{{\rm{j}}}_{Z}}^{\ast (2)}({\boldsymbol{x}})=-\frac{xz}{2({x}^{2}+{y}^{2})}\frac{\partial W}{\partial \theta }(\theta -{\theta }_{{{\rm{j}}}_{\varTheta }},z-{z}_{{{\rm{j}}}_{Z}})+\frac{z}{2}\frac{\partial W}{\partial z}(\theta -{\theta }_{{{\rm{j}}}_{\varTheta }},z-{z}_{{{\rm{j}}}_{Z}})$$

The virtual power of internal forces may be written for any of these virtual fields as:

21 $$\begin{array}{ccc}{P}_{{\rm{i}}{\rm{n}}{\rm{t}}-{{\rm{j}}}_{\varTheta },{{\rm{j}}}_{{\rm{Z}}}}^{\ast ({{\rm{j}}}_{v})}({t}_{{\rm{j}}}) & = & \mathop{\sum }\limits_{{{\rm{i}}}_{Z}=0}^{{{\rm{N}}}_{Z}}\mathop{\sum }\limits_{{{\rm{i}}}_{\varTheta }=0}^{{{\rm{N}}}_{\varTheta }}[\mathop{\sum }\limits_{{\rm{i}}=1}^{3}{{\rm{c}}}_{{{\rm{i}}}_{\varTheta },{{\rm{i}}}_{Z}}^{{\rm{i}}}{\int }_{{\Omega }_{{\rm{w}}{\rm{a}}{\rm{l}}{\rm{l}}}^{{{\rm{i}}}_{\varTheta },{{\rm{i}}}_{Z}}}{J}^{-1}{\psi }^{{\rm{i}}}{\rm{d}}{\rm{e}}{\rm{v}}((\bar{{\boldsymbol{F}}}{{\boldsymbol{G}}}_{{\rm{i}}}{{\bf{a}}}_{{\rm{i}}})\otimes (\bar{{\boldsymbol{F}}}{{\boldsymbol{G}}}_{{\rm{i}}}{{\bf{a}}}_{{\rm{i}}})):{{\boldsymbol{\varepsilon }}}_{{{\rm{j}}}_{\varTheta },{{\rm{j}}}_{Z}}^{\ast ({{\rm{j}}}_{v})}d{\boldsymbol{x}}\\  &  & +{{\rm{c}}}_{{{\rm{i}}}_{\varTheta },{{\rm{i}}}_{Z}}^{4}{\int }_{{\Omega }_{{\rm{w}}{\rm{a}}{\rm{l}}{\rm{l}}}^{{{\rm{i}}}_{\varTheta },{{\rm{i}}}_{Z}}}{J}^{-1}\,{\rm{d}}{\rm{e}}{\rm{v}}((\bar{{\boldsymbol{F}}}{{\boldsymbol{G}}}_{4}){(\bar{{\boldsymbol{F}}}{{\boldsymbol{G}}}_{4})}^{T}):{{\boldsymbol{\varepsilon }}}_{{{\rm{j}}}_{\varTheta },{{\rm{j}}}_{Z}}^{\ast ({{\rm{j}}}_{v})}d{\boldsymbol{x}}\\  &  & +{{\rm{c}}}_{{{\rm{i}}}_{\varTheta },{{\rm{i}}}_{Z}}^{5}{\int }_{{\Omega }_{{\rm{i}}{\rm{m}}{\rm{t}}}^{{{\rm{i}}}_{\varTheta },{{\rm{i}}}_{Z}}}{J}^{-1}\,{\rm{d}}{\rm{e}}{\rm{v}}(\bar{{\boldsymbol{F}}}{\bar{{\boldsymbol{F}}}}^{T}):{{\boldsymbol{\varepsilon }}}_{{{\rm{j}}}_{\varTheta },{{\rm{j}}}_{Z}}^{\ast ({{\rm{j}}}_{v})}d{\boldsymbol{x}}]\end{array}$$

Equation 21 can be written at any time ${t}_{{\rm{j}}}$ at which a measurement was recorded: $1\le {\rm{j}}\le 21$ as we have measurements for 7 pressure states (20, 40, 60, 80, 100, 120 and 140 mmHg) at 3 different axial stretches. Eventually we obtain:

22 $${P}_{{\rm{i}}{\rm{n}}{\rm{t}}\,-{{\rm{j}}}_{\varTheta },{{\rm{j}}}_{Z}}^{\ast ({{\rm{j}}}_{v})}({t}_{{\rm{j}}})=\mathop{\sum }\limits_{{{\rm{i}}}_{Z}=0}^{{{\rm{N}}}_{Z}}\mathop{\sum }\limits_{{{\rm{i}}}_{\varTheta }=0}^{{{\rm{N}}}_{\varTheta }}\mathop{\sum }\limits_{{\rm{i}}=1}^{5}{{\rm{c}}}_{{{\rm{i}}}_{\varTheta },{{\rm{i}}}_{Z}}^{{\rm{i}}}{{\mathcal{A}}}_{{{\rm{j}}}_{\varTheta },{{\rm{j}}}_{Z},{{\rm{i}}}_{\varTheta },{{\rm{i}}}_{Z}}^{{\rm{i}}({{\rm{j}}}_{v})}({t}_{{\rm{j}}})$$

where:

23a $$\begin{array}{c}{{\mathscr{A}}}_{{{\rm{j}}}_{\varTheta },{{\rm{j}}}_{Z},{{\rm{i}}}_{\varTheta },{{\rm{i}}}_{Z}}^{{\rm{i}}({{\rm{j}}}_{v})}({t}_{{\rm{j}}})={\int }_{{\Omega }_{{\rm{w}}{\rm{a}}{\rm{l}}{\rm{l}}}^{{{\rm{i}}}_{\varTheta },{{\rm{i}}}_{Z}}({t}_{{\rm{j}}})}J{({t}_{{\rm{j}}})}^{-1}{\psi }^{{\rm{i}}}({t}_{{\rm{j}}}){\rm{d}}{\rm{e}}{\rm{v}}((\bar{{\boldsymbol{F}}}({t}_{{\rm{j}}}){{\boldsymbol{G}}}_{{\rm{i}}}{{\bf{a}}}_{{\rm{i}}})\otimes (\bar{{\boldsymbol{F}}}({t}_{{\rm{j}}}){{\boldsymbol{G}}}_{{\rm{i}}}{{\bf{a}}}_{{\rm{i}}})):{{\boldsymbol{\varepsilon }}}_{{{\rm{j}}}_{\varTheta },{{\rm{j}}}_{Z}}^{\ast ({{\rm{j}}}_{v})}d{\boldsymbol{x}}\\ {\rm{f}}{\rm{o}}{\rm{r}}\,1\le {\rm{i}}\le 3\end{array}$$

23b $${{\mathscr{A}}}_{{{\rm{j}}}_{\varTheta },{{\rm{j}}}_{Z},{{\rm{i}}}_{\varTheta },{{\rm{i}}}_{Z}}^{4({{\rm{j}}}_{v})}({t}_{{\rm{j}}})={\int }_{{\Omega }_{{\rm{w}}{\rm{a}}{\rm{l}}{\rm{l}}}^{{{\rm{i}}}_{\varTheta },{{\rm{i}}}_{Z}}({t}_{{\rm{j}}})}J{({t}_{{\rm{j}}})}^{-1}{\rm{d}}{\rm{e}}{\rm{v}}((\bar{{\boldsymbol{F}}}({t}_{{\rm{j}}}){{\boldsymbol{G}}}_{4}){(\bar{{\boldsymbol{F}}}({t}_{{\rm{j}}}){{\boldsymbol{G}}}_{4})}^{T}):{{\boldsymbol{\varepsilon }}}_{{{\rm{j}}}_{\varTheta },{{\rm{j}}}_{Z}}^{\ast ({{\rm{j}}}_{v})}d{\boldsymbol{x}}$$

23c $${{\mathscr{A}}}_{{{\rm{j}}}_{\varTheta },{{\rm{j}}}_{Z},{{\rm{i}}}_{\varTheta },{{\rm{i}}}_{Z}}^{5({{\rm{j}}}_{v})}({t}_{{\rm{j}}})={\int }_{{{\Omega }}_{{\rm{imt}}}^{{{\rm{i}}}_{\varTheta },{{\rm{i}}}_{Z}}({t}_{{\rm{j}}})}J{({t}_{{\rm{j}}})}^{-1}{\rm{dev}}(\bar{{\boldsymbol{F}}}({t}_{{\rm{j}}})\bar{{\boldsymbol{F}}}{({t}_{{\rm{j}}})}^{T}):{{\boldsymbol{\varepsilon }}}_{{{\rm{j}}}_{\varTheta },{{\rm{j}}}_{Z}}^{\ast ({{\rm{j}}}_{v})}d{\boldsymbol{x}}$$

Reorganizing indices ${{\rm{j}}}_{\varTheta },{{\rm{j}}}_{Z},{{\rm{j}}}_{v}$ and j as a single index j (varying between 1 and 28N_*Θ*_N_*Z*_) and indices ${{\rm{i}}}_{\varTheta },{{\rm{i}}}_{Z}$ and i as a single index i (varying between 1 and 5N_*Θ*_N_*Z*_), Eq. 22 can be rewritten:

24 $${P}_{{\rm{i}}{\rm{n}}{\rm{t}}}^{\ast {\rm{j}}}=\mathop{\sum }\limits_{{\rm{i}}=1}^{5{{\rm{N}}}_{\varTheta }{{\rm{N}}}_{Z}}{{\mathscr{A}}}_{{\rm{j}}{\rm{i}}}{{\rm{c}}}_{{\rm{i}}}$$

where the ${{\mathscr{A}}}_{{\rm{ji}}}$ terms, derived using Eq. 23a-c, depend only on the set of unknown nonlinear parameters ${\Xi }^{{\rm{i}}}$, further denoted as vector ${\boldsymbol{\Xi }}$. All integrals in Eq. 23a-c are computed by summing the values taken by each function at the centroid of each ${{\rm{N}}}_{{\Omega }_{{\rm{wall}}}}$ tetrahedron multiplied by the volume of each tetrahedron while meshing the whole domain of interest.

For any index j, the value of the virtual power of external forces, denoted ${P}_{{\rm{ext}}}^{\ast {\rm{j}}}$, is deduced from the measured values of the intraluminal pressure and axial force, introducing the expressions of Eq. 17 into the second term of Eq. 14. Again, integrals are computed by summing the values taken by each function at the centroid of each ${{\rm{N}}}_{{\rm{out}}}$ triangular facet times the volume of each facet meshing the outer surface.

Rewriting the sets of ${P}_{{\rm{ext}}}^{\ast {\rm{j}}}$ and ${{\rm{c}}}_{{\rm{i}}}$ values as vectors: $\{{{\boldsymbol{P}}}_{{\rm{int}}}^{\ast }\}$, $\{{{\boldsymbol{P}}}_{{\rm{ext}}}^{\ast }\}$ and $\{{\bf{c}}\}$ and the sets of ${{\mathscr{A}}}_{{\rm{ji}}}^{\ast }$ values as a rectangular matrix denoted $[\boldsymbol{\mathscr{A}}]$ (with dimensions $28{{\rm{N}}}_{\varTheta }{{\rm{N}}}_{Z}$ × $5{{\rm{N}}}_{\varTheta }{{\rm{N}}}_{Z}$), Eq. 23 can be written in matrix form as

25 $$[\boldsymbol{\mathscr{A}}({\boldsymbol{\Xi }})]\{{\bf{c}}\}=\{{{\boldsymbol{P}}}_{{\rm{ext}}}^{\ast }\}.$$

The system is solved in the least squares sense. For a given vector of nonlinear parameters ${\boldsymbol{\Xi }}$, the solution is

26 $$\{{\bf{c}}\}={[{[\boldsymbol{\mathscr{A}}({\boldsymbol{\Xi }})]}^{T}[\boldsymbol{\mathscr{A}}({\boldsymbol{\Xi }})]]}^{-1}{[\boldsymbol{\mathscr{A}}({\boldsymbol{\Xi }})]}^{T}\{{{\boldsymbol{P}}}_{{\rm{ext}}}^{\ast }\}.$$

The optimal set of nonlinear parameters is obtained by minimizing the following cost function:

27 $$\begin{array}{c} {\mathcal H} ({\boldsymbol{\Xi }})={\{{{\boldsymbol{P}}}_{{\rm{ext}}}^{\ast }\}}^{T}{[[\boldsymbol{\mathscr{A}}({\boldsymbol{\Xi }})]{[{[\boldsymbol{\mathscr{A}}({\boldsymbol{\Xi }})]}^{T}[\boldsymbol{\mathscr{A}}({\boldsymbol{\Xi }})]]}^{-1}{[\boldsymbol{\mathscr{A}}({\boldsymbol{\Xi }})]}^{T}-{\boldsymbol{I}}]}^{{\boldsymbol{T}}}\\ \,[[\boldsymbol{\mathscr{A}}({\boldsymbol{\Xi }})]{[{[\boldsymbol{\mathscr{A}}({\boldsymbol{\Xi }})]}^{T}[\boldsymbol{\mathscr{A}}({\boldsymbol{\Xi }})]]}^{-1}{[\boldsymbol{\mathscr{A}}({\boldsymbol{\Xi }})]}^{T}-{\boldsymbol{I}}]\{{{\boldsymbol{P}}}_{{\rm{ext}}}^{\ast }\}\end{array}$$

with bounds assigned to each nonlinear parameter^5,22^ and the minimization performed in MATLAB using a genetic algorithm^5^.

### Histology

Following the three-stage mechanical testing and imaging protocol, the aortic segments were fixed in formalin and stored in 70% EtOH until all specimens could be embedded in paraffin, sectioned, and stained together. Sections were obtained at three representative cross sections denoted S1, S2 and S3. S2 is approximately located at mid-length of each sample, whereas S1 is approximately at 1/4 and S3 is approximately at 3/4.

Standard Movat’s Pentachrome staining (MOV) was used to assess the organization of the extracellular matrix, with thrombus (fibrin) staining red, elastin black, and collagen yellow-grey. An Olympus BX/51 microscope (Olympus Inc., Center Valley, PA), equipped with an Olympus DP70 digital camera, was used to acquire images using consistent settings. Digital images were then analyzed using custom MATLAB software^22^ to quantify the average mass fractions of the primary structural constituents: elastic fibers, SMCs, fibrin, and fibrillar collagens (see Fig. S2). Mass fractions of each constituent were qualitatively and quantitatively related to the local material properties obtained with the inverse method described previously.

In addition, for comparison with local mechanical property measurements, histological images were divided into 125 partitions (25 circumferential × 5 radial; see Figs. 6–8). Colorimetric analysis within each partition was used to estimate local area fractions (i.e., wall composition). For the registration of histological and mechanical property images, histological wall thickness ${T}_{hist}$ was estimated for each circumferential and axial position (*θ*, *z*) using an Eulerian solution to a pair of linear partial differential equations solved over the histological domain, as described previously^22^. Relative wall thickness (local thickness divided by maximum thickness) was then deduced for every (*θ*, *z*) position. The local thickness was also estimated from the mesh of the artery at every (*θ*, *z*) position and was denoted ${T}_{mesh}$. A cost function was defined as:

$$C(\theta ,z)={\int }_{0}^{2\pi }{\left(\frac{{T}_{hist}(\vartheta )}{max({T}_{hist})},-,\frac{{T}_{mesh}(\vartheta +\theta ,z)}{max({T}_{mesh})}\right)}^{2}d\vartheta $$

and the (*θ*, *z*) values that minimize *C* (the difference in relative thickness between histological images and the arterial mesh) were identified with the Nelder Mead minimization algorithm in MATLAB. The optimal circumferential and axial positions for cross-sections S1, S2 and S3 were registered in order to have the best agreement between the relative thickness of histological images and of mechanical reconstructions. Using this approach, the cross section at which mechanical properties corresponding to the given histological image should be read was obtained, and the rotation that had to be applied for the mechanical reconstructions to be registered with the histological image was identified.

### Statistics

Correlation analysis was used to evaluate the strength of relationships between measured variables. Linear correlations were evaluated by the Pearson correlation coefficient *R* and non-parametric correlations were evaluated by the Spearman rank correlation coefficient *ρ*. For all correlation analyses, a value of *P* < 0.05 was considered significant.

## Supplementary information

Supplementary Information.

Supplementary Information 2.
