# Supplementary material for: Multimodality Imaging-Based Characterization of Regional Material Properties in a Murine Model of Aortic Dissection
Source: Sci Rep. 2020 Jun 8;10:9244. doi: 10.1038/s41598-020-65624-7 (PMC7280301; doi:10.1038/s41598-020-65624-7)
Supplement: Supplementary file 1 — Supplementary Information. [file 41598_2020_65624_MOESM1_ESM.pdf]

Supplementary Information

Multimodality Imaging-Based Characterization of Regional Material Properties in a Murine Model of Aortic Dissection

Matthew R. Bersi[1,2], Víctor Acosta Santamaría[3], Karl Marback[1], Paolo Di Achille[1], Evan H. Phillips[4], Craig J. Goergen[4], Jay D. Humphrey[1,5], Stéphane Avril[3]

[1]Department of Biomedical Engineering, Yale University, New Haven, CT, USA
[2]Department of Biomedical Engineering, Vanderbilt University, Nashville, TN, USA
[3]Mines Saint-Etienne, University of Lyon, University Jean Monnet, INSERM, Saint-Etienne, France
[4]Weldon School of Biomedical Engineering, Purdue University, West Lafayette, IN, USA
[5]Vascular Biology and Therapeutics Program, Yale School of Medicine, New Haven, CT, USA

Corresponding author:

Stéphane Avril
Mines Saint-Etienne,
F-42023 Saint Etienne, France
avril@emse.fr

Supplemental Methods

The basic principle of DVC is the following. In the reference configuration the OCT image can be represented by the intensity level function $\mathrm{f}(\mathrm{x},\mathrm{y},\mathrm{z})$, and in the deformed state by the intensity level function $\mathrm{g}(\mathrm{x},\mathrm{y},\mathrm{z})$, where $(\mathrm{x},\mathrm{y},\mathrm{z})$ represents the coordinates. Functions f and g are reconstructed continuously for any $(\mathrm{x},\mathrm{y},\mathrm{z})$ position from the intensity level of each voxel using third-order spline interpolation. Denoting $(\mathrm{u},\mathrm{v},\mathrm{w})$ as the displacement field between the deformed configuration and the reference configuration, the conservation of intensity can be assumed as $\mathrm{f}(\mathrm{x},\mathrm{y},\mathrm{z}) = \mathrm{g}(\mathrm{x}+\mathrm{u},\mathrm{y}+\mathrm{v},\mathrm{z}+\mathrm{w})$. The objective is to find the field $(\mathrm{u},\mathrm{v},\mathrm{w})$ maximizing the cross-correlation between $\mathrm{f}(\mathrm{x},\mathrm{y},\mathrm{z})$ and $\mathrm{g}(\mathrm{x}+\mathrm{u},\mathrm{y}+\mathrm{v},\mathrm{z}+\mathrm{w})$.

The domain of interest is discretized in correlation subvolumes Ω_{corr} and for each subvolume, the displacement field is assumed to be linear such as:

$$\begin{Bmatrix} \mathrm{u}(x,y,z) \\ \mathrm{v}(x,y,z) \\ \mathrm{w}(x,y,z) \end{Bmatrix} = \begin{Bmatrix} \mathrm{u}_0 \\ \mathrm{v}_0 \\ \mathrm{w}_0 \end{Bmatrix} + \begin{bmatrix} \varepsilon_{xx} & \varepsilon_{xy}+\omega_{xy} & \varepsilon_{xz}-\omega_{xz} \\ \varepsilon_{xy}-\omega_{xy} & \varepsilon_{yy} & \varepsilon_{yz}+\omega_{yz} \\ \varepsilon_{xz}+\omega_{xz} & \varepsilon_{yz}-\omega_{yz} & \varepsilon_{zz} \end{bmatrix} \begin{Bmatrix} x-x_0 \\ y-y_0 \\ z-z_0 \end{Bmatrix} \qquad \text{(A1)}$$

where (x_0, y_0, z_0) denote the centre of a subvolume, $(\mathrm{u}_0, \mathrm{v}_0, \mathrm{w}_0)$ denote the displacement at the center of the subvolume and ε_{xx}, ε_{yy}, ε_{zz}, ε_{yz}, ε_{xz}, ε_{xy}, ω_{yz}, ω_{xz}, ω_{xy} are regularization parameters permitting a more precise description of the subvolume deformation. Although they may characterize the average strain and rotation across the subvolume, they are not used further as deformation gradients are obtained after fitting the displacement with polynomial shape functions. The subvolumes are defined with an overlapping of 75%, meaning that the centre of two adjacent subvolumes are separated by 1/4 of the edge size of each subvolume.

For each subvolume, u_0, v_0, w_0, ε_{xx}, ε_{yy}, ε_{zz}, ε_{yz}, ε_{xz}, ε_{xy}, ω_{yz}, ω_{xz} and ω_{xy} , further denoted as vector $\{\gamma_0\}$, are found by maximizing the cross-correlation coefficient defined such as:

$$\mathcal{C}(\{\gamma_0\}) = \frac{\iiint_{\Omega_{\mathrm{corr}}} \mathrm{f(x,y,z)g\big(x+u(}\{\gamma_0\}\mathrm{),y+v(}\{\gamma_0\}\mathrm{),z+w(}\{\gamma_0\}\mathrm{)\big)d}x\mathrm{d}y\mathrm{d}z}{\iiint_{\Omega_{\mathrm{corr}}} \mathrm{f(x,y,z)d}x\mathrm{d}y\mathrm{d}z \iiint_{\Omega_{\mathrm{corr}}} \mathrm{g(x,y,z)d}x\mathrm{d}y\mathrm{d}z} \qquad \text{(A2)}$$

Preliminary investigations with our OCT images showed that higher cross-correlation coefficients could be obtained by performing the minimization directly (the other option being to perform it in the Fourier space). In that case, it is assumed that:

$$\mathrm{g}(\mathrm{x}+\mathrm{u}+\delta u,\mathrm{y}+\mathrm{v}+\delta v,\mathrm{z}+\mathrm{w}+\delta w) = \mathrm{g}(\mathrm{x}+\mathrm{u},\mathrm{y}+\mathrm{v},\mathrm{z}+\mathrm{w}) +$$
$$\delta\mathrm{u}\frac{\partial g}{\partial x}(\mathrm{x}+\mathrm{u},\mathrm{y}+\mathrm{v},\mathrm{z}+\mathrm{w}) + \delta\mathrm{v}\frac{\partial g}{\partial y}(\mathrm{x}+\mathrm{u},\mathrm{y}+\mathrm{v},\mathrm{z}+\mathrm{w}) + \delta\mathrm{w}\frac{\partial g}{\partial z}(\mathrm{x}+\mathrm{u},\mathrm{y}+\mathrm{v},\mathrm{z}+\mathrm{w}) \qquad \text{(A3)}$$

$$\mathrm{g}(\mathrm{x}+\mathrm{u}+\delta u,\mathrm{y}+\mathrm{v}+\delta v,\mathrm{z}+\mathrm{w}+\delta w)=\mathrm{g}(\mathrm{x}+\mathrm{u},\mathrm{y}+\mathrm{v},\mathrm{z}+\mathrm{w})+\begin{Bmatrix}\frac{\partial g}{\partial x}(\mathrm{x}+\mathrm{u},\mathrm{y}+\mathrm{v},\mathrm{z}+\mathrm{w})\\ \frac{\partial g}{\partial y}(\mathrm{x}+\mathrm{u},\mathrm{y}+\mathrm{v},\mathrm{z}+\mathrm{w})\\ \frac{\partial g}{\partial z}(\mathrm{x}+\mathrm{u},\mathrm{y}+\mathrm{v},\mathrm{z}+\mathrm{w})\end{Bmatrix}.\begin{Bmatrix}\delta u_0\\ \delta v_0\\ \delta w_0\end{Bmatrix}+$$

$$\begin{Bmatrix}\frac{\partial g}{\partial x}(\mathrm{x}+\mathrm{u},\mathrm{y}+\mathrm{v},\mathrm{z}+\mathrm{w})\\ \frac{\partial g}{\partial y}(\mathrm{x}+\mathrm{u},\mathrm{y}+\mathrm{v},\mathrm{z}+\mathrm{w})\\ \frac{\partial g}{\partial z}(\mathrm{x}+\mathrm{u},\mathrm{y}+\mathrm{v},\mathrm{z}+\mathrm{w})\end{Bmatrix}.\begin{bmatrix}\delta\varepsilon_{xx} & \delta\varepsilon_{xy}+\delta\omega_{xy} & \delta\varepsilon_{xz}-\delta\omega_{xz}\\ \delta\varepsilon_{xy}-\delta\omega_{xy} & \delta\varepsilon_{yy} & \delta\varepsilon_{yz}+\delta\omega_{yz}\\ \delta\varepsilon_{xz}+\delta\omega_{xz} & \delta\varepsilon_{yz}-\delta\omega_{yz} & \delta\varepsilon_{zz}\end{bmatrix}\begin{Bmatrix}x-x_0\\ y-y_0\\ z-z_0\end{Bmatrix} \qquad \text{(A4)}$$

$$\mathrm{g}(\mathrm{x}+\mathrm{u}+\delta u,\mathrm{y}+\mathrm{v}+\delta v,\mathrm{z}+\mathrm{w}+\delta w)=$$

$$\begin{Bmatrix}\frac{\partial g}{\partial x}(\mathrm{x}+\mathrm{u},\mathrm{y}+\mathrm{v},\mathrm{z}+\mathrm{w})\\ \frac{\partial g}{\partial y}(\mathrm{x}+\mathrm{u},\mathrm{y}+\mathrm{v},\mathrm{z}+\mathrm{w})\\ \frac{\partial g}{\partial z}(\mathrm{x}+\mathrm{u},\mathrm{y}+\mathrm{v},\mathrm{z}+\mathrm{w})\\ \frac{\partial g}{\partial x}(\mathrm{x}+\mathrm{u},\mathrm{y}+\mathrm{v},\mathrm{z}+\mathrm{w}).(x-x_0)\\ \frac{\partial g}{\partial y}(\mathrm{x}+\mathrm{u},\mathrm{y}+\mathrm{v},\mathrm{z}+\mathrm{w}).(y-y_0)\\ \frac{\partial g}{\partial z}(\mathrm{x}+\mathrm{u},\mathrm{y}+\mathrm{v},\mathrm{z}+\mathrm{w}).(z-z_0)\\ \frac{\partial g}{\partial y}(\mathrm{x}+\mathrm{u},\mathrm{y}+\mathrm{v},\mathrm{z}+\mathrm{w}).(z-z_0)+\frac{\partial g}{\partial z}(\mathrm{x}+\mathrm{u},\mathrm{y}+\mathrm{v},\mathrm{z}+\mathrm{w}).(y-y_0)\\ \frac{\partial g}{\partial x}(\mathrm{x}+\mathrm{u},\mathrm{y}+\mathrm{v},\mathrm{z}+\mathrm{w}).(z-z_0)+\frac{\partial g}{\partial z}(\mathrm{x}+\mathrm{u},\mathrm{y}+\mathrm{v},\mathrm{z}+\mathrm{w}).(x-x_0)\\ \frac{\partial g}{\partial x}(\mathrm{x}+\mathrm{u},\mathrm{y}+\mathrm{v},\mathrm{z}+\mathrm{w}).(y-y_0)+\frac{\partial g}{\partial y}(\mathrm{x}+\mathrm{u},\mathrm{y}+\mathrm{v},\mathrm{z}+\mathrm{w})(x-x_0)\\ \frac{\partial g}{\partial y}(\mathrm{x}+\mathrm{u},\mathrm{y}+\mathrm{v},\mathrm{z}+\mathrm{w}).(z-z_0)-\frac{\partial g}{\partial z}(\mathrm{x}+\mathrm{u},\mathrm{y}+\mathrm{v},\mathrm{z}+\mathrm{w}).(y-y_0)\\ \frac{\partial g}{\partial x}(\mathrm{x}+\mathrm{u},\mathrm{y}+\mathrm{v},\mathrm{z}+\mathrm{w}).(z-z_0)-\frac{\partial g}{\partial z}(\mathrm{x}+\mathrm{u},\mathrm{y}+\mathrm{v},\mathrm{z}+\mathrm{w}).(x-x_0)\\ \frac{\partial g}{\partial x}(\mathrm{x}+\mathrm{u},\mathrm{y}+\mathrm{v},\mathrm{z}+\mathrm{w}).(y-y_0)-\frac{\partial g}{\partial y}(\mathrm{x}+\mathrm{u},\mathrm{y}+\mathrm{v},\mathrm{z}+\mathrm{w}).(x-x_0)\end{Bmatrix}.\begin{Bmatrix}\delta u_0\\ \delta v_0\\ \delta w_0\\ \delta\varepsilon_{xx}\\ \delta\varepsilon_{yy}\\ \delta\varepsilon_{zz}\\ \delta\varepsilon_{yz}\\ \delta\varepsilon_{xz}\\ \delta\varepsilon_{xy}\\ \delta\omega_{yz}\\ \delta\omega_{xz}\\ \delta\omega_{xy}\end{Bmatrix} \qquad \text{(A5)}$$

$$\mathrm{g}(\mathrm{x}+\mathrm{u}+\delta u,\mathrm{y}+\mathrm{v}+\delta v,\mathrm{z}+\mathrm{w}+\delta w)=\{\nabla g\}.\{\delta\gamma_0\} \qquad \text{(A6)}$$

$$\mathcal{C}(\mathrm{x}+\mathrm{u}+\delta u,\mathrm{y}+\mathrm{v}+\delta v,\mathrm{z}+\mathrm{w}+\delta w)=\mathcal{C}(\mathrm{x}+\mathrm{u},\mathrm{y}+\mathrm{v},\mathrm{z}+\mathrm{w})+\frac{\iiint_{\Omega_{\mathrm{corr}}}\mathrm{f}(\mathrm{x,y,z})\{\nabla g(\mathrm{x+u,y+v,z+w})\}.\{\delta{\gamma_0}^{k}\}\mathrm{d}x\mathrm{d}y\mathrm{d}z}{\iiint_{\Omega_{\mathrm{corr}}}\mathrm{f}(\mathrm{x,y,z})\mathrm{d}x\mathrm{d}y\mathrm{d}z\iiint_{\Omega_{\mathrm{corr}}}\mathrm{g}(\mathrm{x,y,z})\mathrm{d}x\mathrm{d}y\mathrm{d}z}$$

(A7)

$$\mathcal{C}(\mathrm{x}+\mathrm{u}+\delta u,\mathrm{y}+\mathrm{v}+\delta v,\mathrm{z}+\mathrm{w}+\delta w)=\mathcal{C}(\mathrm{x}+\mathrm{u},\mathrm{y}+\mathrm{v},\mathrm{z}+\mathrm{w})+\{\nabla\mathcal{C}\}.\{\delta\gamma_0\} \qquad \text{(A8)}$$

Then the maximization of $\mathcal{C}$ is performed using a Newton algorithm such as:

$$\{{\gamma_0}^{k+1}\} = \{{\gamma_0}^{k}\} - \frac{1}{\nabla\mathcal{C}(\mathrm{x+u,y+v,z+w})} \frac{\iiint_{\Omega_{\mathrm{corr}}} \mathrm{f(x,y,z)g}\left(\mathrm{x+u}^{k}\mathrm{,y+v}^{k}\mathrm{,z+w}^{k}\right)\mathrm{d}x\mathrm{d}y\mathrm{d}z}{\iiint_{\Omega_{\mathrm{corr}}} \mathrm{f(x,y,z)d}x\mathrm{d}y\mathrm{d}z \iiint_{\Omega_{\mathrm{corr}}} \mathrm{g(x,y,z)d}x\mathrm{d}y\mathrm{d}z} \quad \text{(A9)}$$

As it can be seen in Eq. A9, a sufficient contrast is required in order to determine a displacement vector with enough accuracy and reduce the measurements uncertainties, as the terms $\nabla\mathcal{C}$, which is defined from the image intensity gradients, is at the denominator.

Supplemental Figures

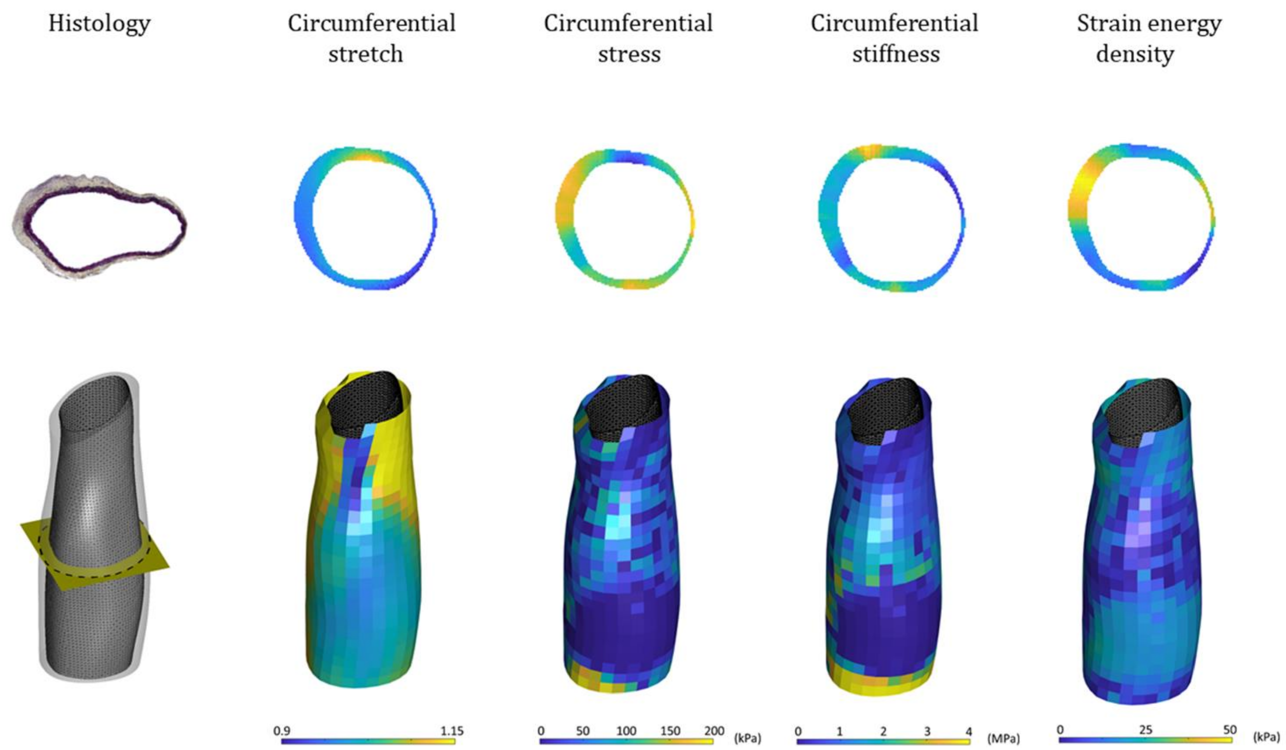

Fig S1. Analysis and results obtained for the non-dissected SAA sample from mouse M1, which did not develop a thrombus. Shown are distributions of circumferential stretch, circumferential stress and circumferential material stiffness at a distending pressure of 140 mmHg at the *in vivo* value of axial stretch λ^{iv}. Elastic stored energy is shown at a pressure of 80 mmHg at the *in vivo* value of axial stretch λ^{iv}. Note the histological cross-section (left; MOV), in which thrombus (fibrin) stains red (absent here), elastin black, and collagen brown-grey.

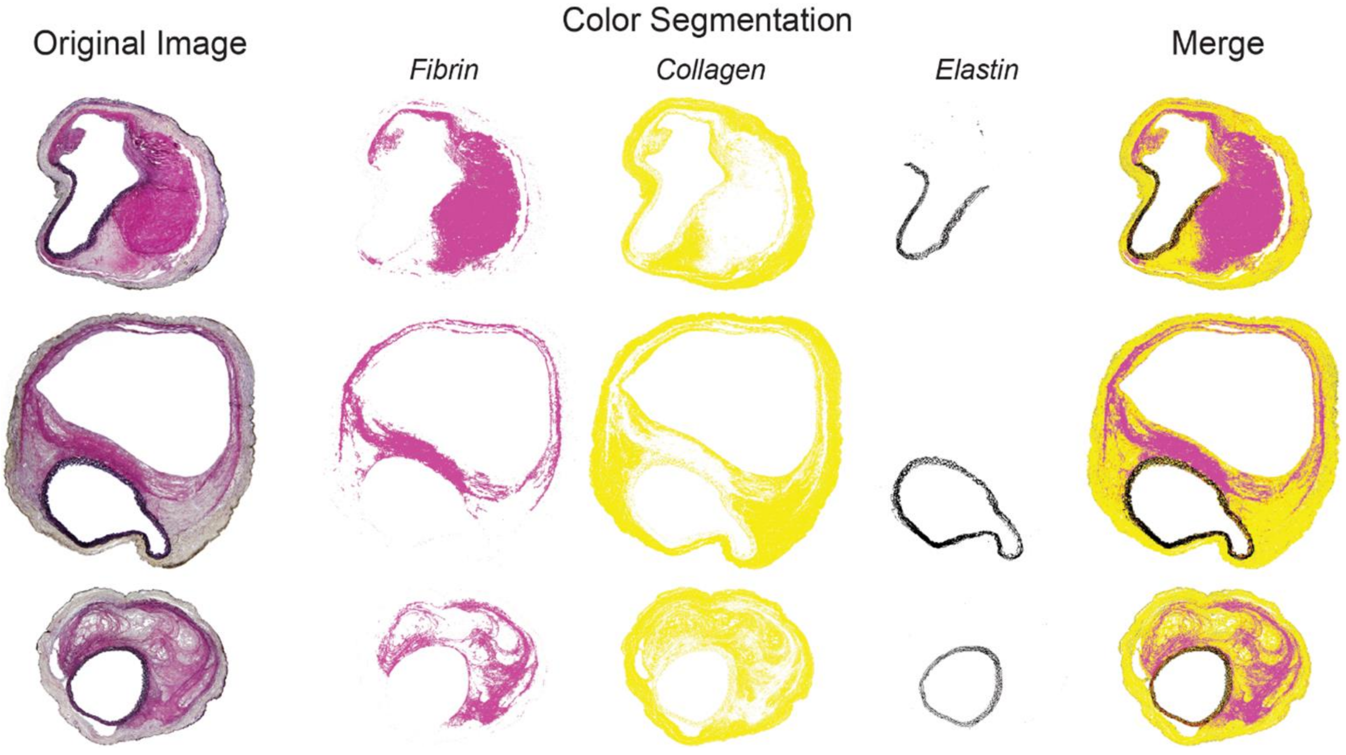

Fig S2. Representative histological images illustrating color decomposition based on a HSL-based colorimetric analysis. Note the ability to separate the pink/red pixels of fibrin, the brown/grey pixels of collagen, and the black pixels of elastin. Pseudo-colored images (right) can be compared with original images (left) to visualize color classification.

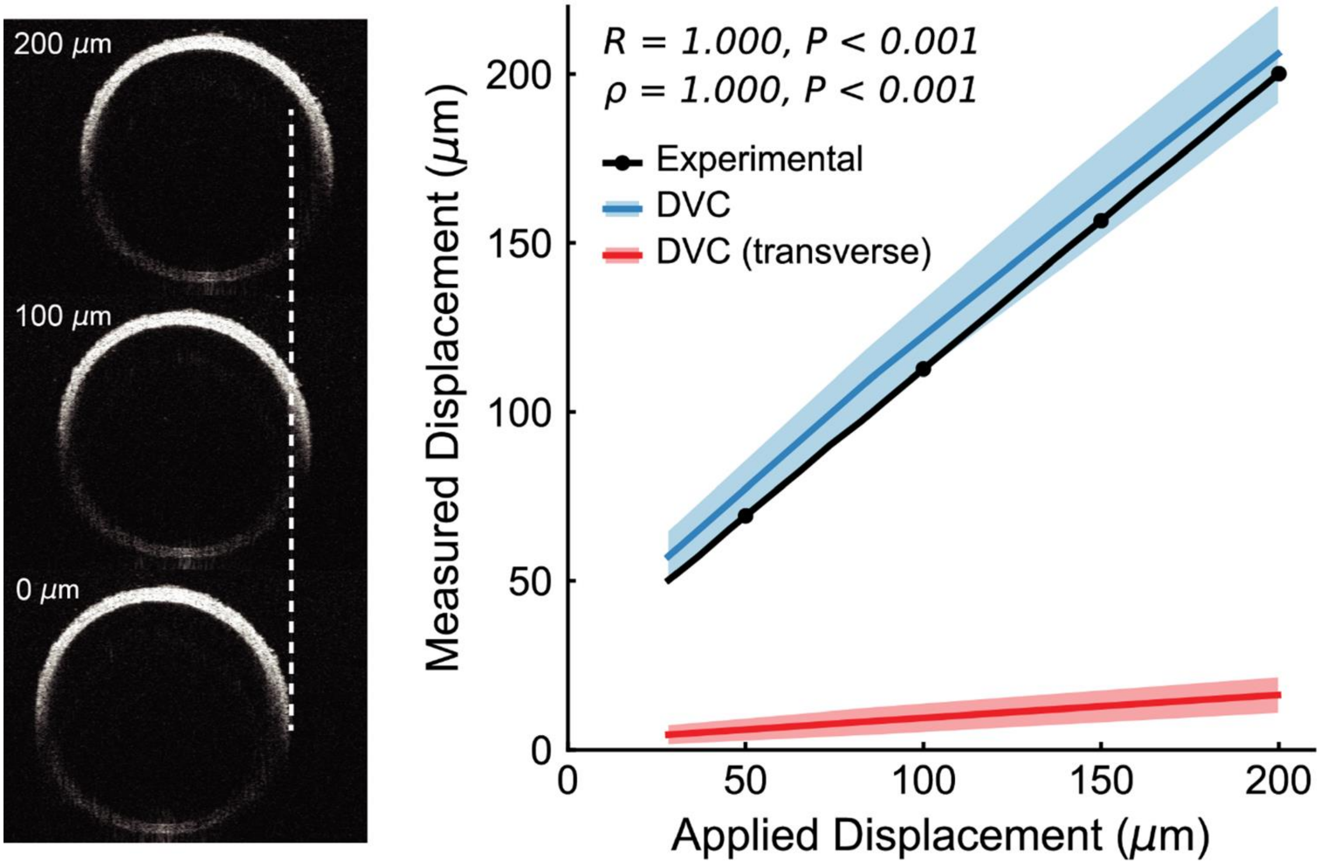

Fig S3. Comparison between experimentally applied displacements (black) and the corresponding OCT-DVC displacement measurements for a SAA segment that experienced rigid body motions. Displacement in the direction of translation (blue) shows a good agreement with the experimentally applied motion; note the minimal error in the transverse direction (red).

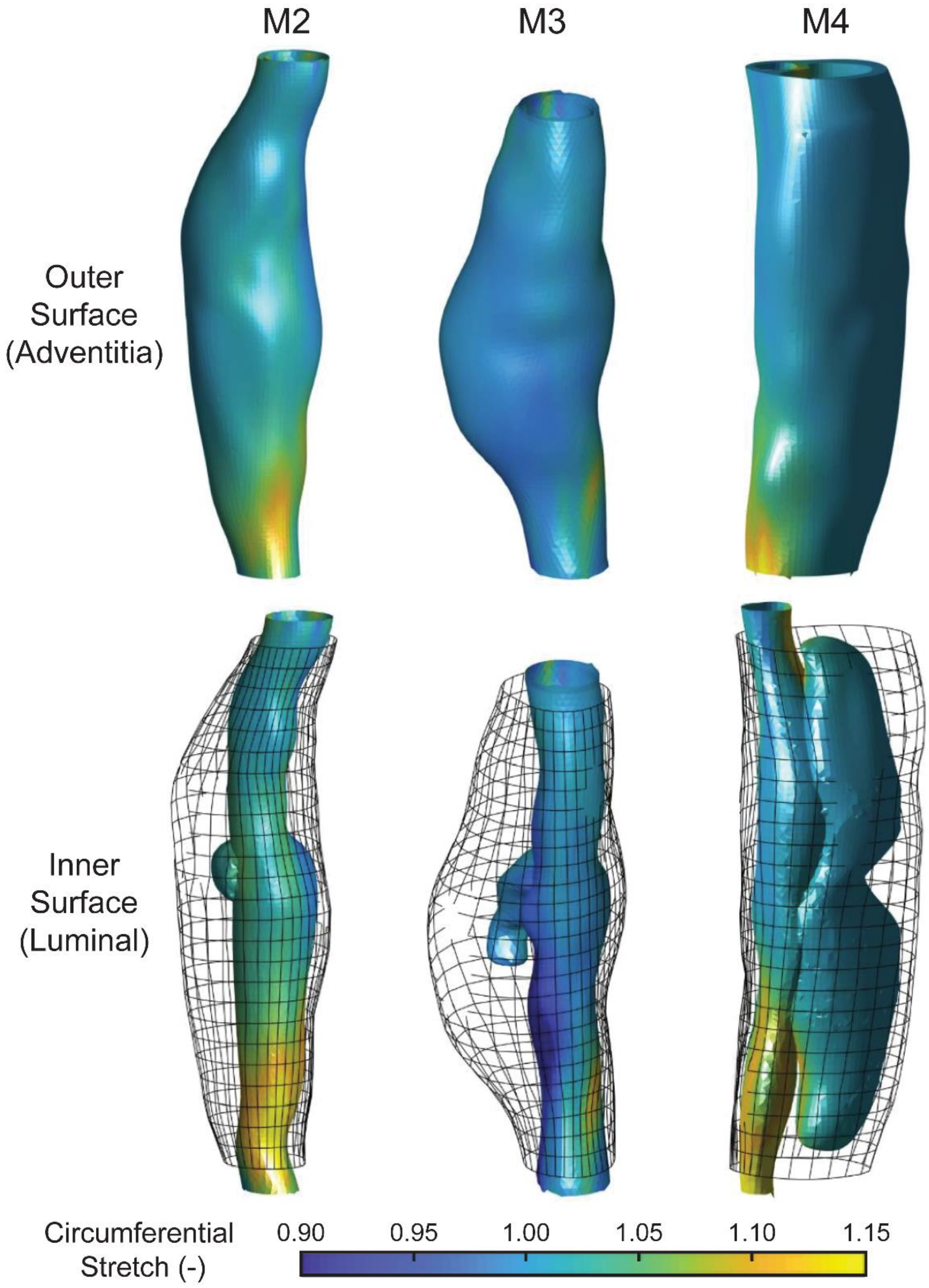

Fig S4. Maps of circumferential stretch at the outer (adventitial; top row) and inner (luminal; bottom row) surface of the dissected samples from M2 (first column), M3 (second column), and M4 (third column). The distribution of circumferential stretch is represented at a distending pressure of 140 mmHg and the *in vivo* axial stretch λ^{iv}. Wireframe in the bottom row denotes the location of the outer wall. Data for the inner and outer surfaces is displayed at the same scale.

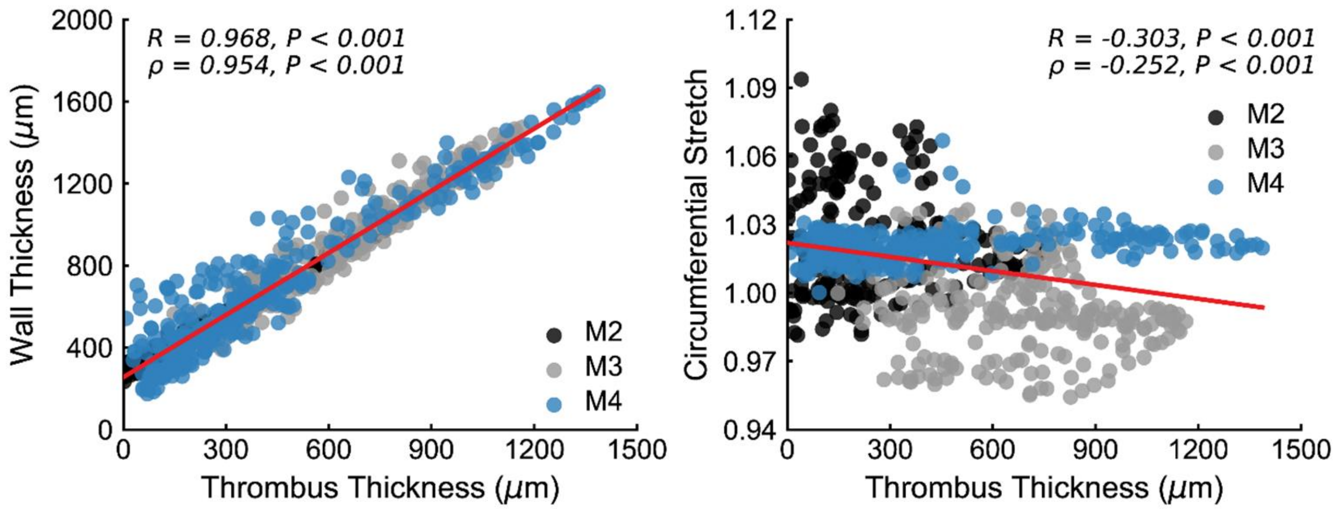

Fig. S5. Comparison of total wall thickness with thrombus thickness (from Fig. 2) reveals a strong positive correlation, and comparison of circumferential stretch at the outer adventitial surface with thrombus thickness revealed a slight negative correlation (particularly in samples M2 and M3). Together, this suggests that the presence of intramural thrombus has a significant effect on wall thickness and has a slight impact on the mechanical response of the wall.

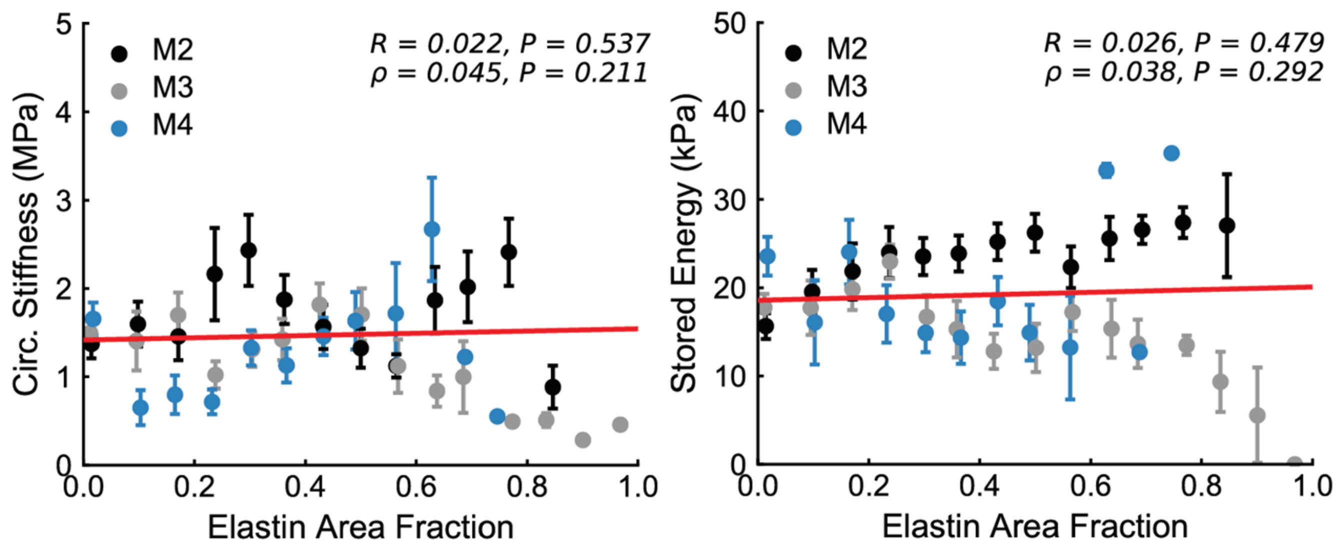

Fig. S6. Comparisons between local values of circumferential stiffness and stored energy and elastin area fractions reveal no apparent trends. This suggests elastin has a minimal effect on the overall mechanical response which is instead dominated by collagen and fibrin.
