# Supplementary material for: Multimodality Imaging-Based Characterization of Regional Material Properties in a Murine Model of Aortic Dissection
Source: Sci Rep. 2020 Jun 8;10:9244. doi: 10.1038/s41598-020-65624-7 (PMC7280301; doi:10.1038/s41598-020-65624-7)
Supplement: Supplementary file 2 — Supplementary Information 2. [file 41598_2020_65624_MOESM2_ESM.docx]

**Multimodality Imaging-Based Characterization of Regional Material Properties in a Murine Model of Aortic Dissection**

Matthew R. Bersi^1,2^, Víctor Acosta Santamaría^3^, Karl Marback^1^, Paolo Di Achille^1^, Evan H.

Phillips^4^, Craig J. Goergen^4^, Jay D. Humphrey^1,5^, Stéphane Avril^3^

^1^Department of Biomedical Engineering, Yale University, New Haven, CT, USA

^2^Department of Biomedical Engineering, Vanderbilt University, Nashville, TN, USA

^3^Mines Saint-Etienne, University of Lyon, University Jean Monnet, INSERM, Saint-Etienne, France

^4^Weldon School of Biomedical Engineering, Purdue University, West Lafayette, IN, USA

^5^Vascular Biology and Therapeutics Program, Yale School of Medicine, New Haven, CT, USA

# Corresponding author:

Stéphane Avril

Mines Saint-Etienne,

F-42023 Saint Etienne, France

avril@emse.fr

Keywords: INVERSE METHOD, MATERIAL HETEROGENEITY, structure-function, AORTIC ANEURYSM, AORTIC DiSSECTION, AngIOTENSIN, mOUSE
